# Practical Enantioselective Approach to 3‐Amino‐2‐Hydroxy Acids and Application to the Synthesis of Natural Products

**DOI:** 10.1002/chir.70115

**Published:** 2026-06-17

**Authors:** Marilena Caporale, Giulia Marsico, Ernesto Santoro, Patrizia Scafato, Stefano Superchi

**Affiliations:** ^1^ Department of Basic and Applied Sciences University of Basilicata Potenza Italy

**Keywords:** asymmetric dihydroxylation, enantioselective synthesis, marine products, perthamide C

## Abstract

A practical approach to optically active *erythro* 3‐amino‐2‐hydroxy acids has been developed and applied to the enantioselective synthesis of 3‐phenylisoserine and naturally occurring 3‐amino‐2‐hydroxy‐6‐methylheptanoic acid (AHMHA), a nonproteinogenic amino acid found in the marine cyclic oligopeptide perthamide C. The proposed synthetic methodology requires the enantioselective Sharpless *syn*‐dihydroxylation of (*E*)‐α,β‐unsaturated esters followed by regioselective and stereoselective Mitsunobu azidation on the β‐hydroxy group. Subsequent azide hydrogenation and ester hydrolysis provide the desired *erythro* 3‐amino‐2‐hydroxy acids in high enantiopurity and overall yield. The proposed approach to AHMHA was more efficient and direct compared to the one previously reported in the literature. The absolute configuration of the diol precursor of AHMHA was assigned by ECD analysis of its biphenyl dioxolane, thereby also confirming the absolute configuration of the natural AHMHA.

## Graphical abstract

A practical and straightforward approach to optically active *erythro* 3‐amino‐2‐hydroxy acids and its application to the enantioselective synthesis of 3‐phenylisoserine and of the marine natural product 3‐amino‐2‐hydroxy‐6‐methylheptanoic acid is reported.

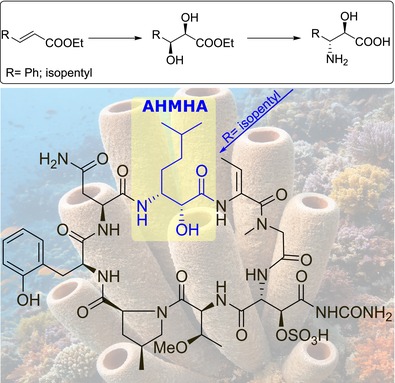

## 1 Introduction

**SCHEME 1:**
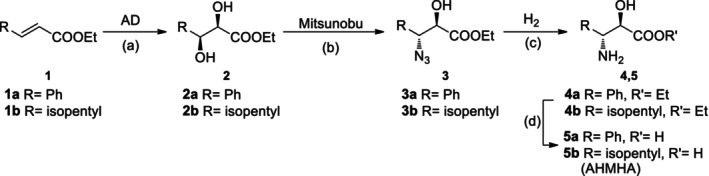
General synthetic approach to *erythro* 3‐amino‐2‐hydroxy ester. Reagents and conditions: (a) AD‐mix‐α, *t‐*BuOH/H_2_O, MeSO_4_NH_2_, 0°C; (b) Me_3_SiN_3_, PPh_3_, DIAD, THF, 0°C to rt; (c) H_2_, Pd/C, EtOH, rt, (d) LiOH 2 M, THF, reflux.

The interest in natural products chemistry is particularly motivated by their chemical diversity and the significant opportunity to discover novel molecules for the development of new classes of pharmaceuticals. The structural elucidation of natural products often requires their total synthesis. Therefore, the discovery of new synthetic approaches to gain access to structural motifs particularly widespread in natural products and bioactive drugs is of paramount importance in organic chemistry. An interesting example is constituted by chiral 3‐amino‐2‐hydroxy acids, which represent a valuable class of compounds due to their unique structural features and broad range of biological and synthetic applications. These molecules also serve as key intermediates for the synthesis of natural products, pharmaceuticals, and peptidomimetics, and they often exhibit significant biological activity on their own. In fact, the chiral 3‐amino‐2‐hydroxy acid moiety is quite common in natural products having therapeutical applications, like peptidomimetic protease inhibitors microginin [1], bestatin [2, 3], and amastatin [4, 5], antibacterial edeine [6], and antitumor agent paclitaxel (Taxol) [7]. Moreover, the presence of a hydroxyl and an amino stereogenic functionality adjacent to each other makes them versatile building blocks for the design of novel bioactive molecules and catalysts. For these reasons, the development of efficient and stereoselective synthetic routes to 3‐amino‐2‐hydroxy acids remains an important challenge in modern organic chemistry. Several approaches have been reported for the diastereoselective and enantioselective synthesis of such moieties, including chiral pool [8, 9], asymmetric aldol reactions [10], epoxide opening [11], Sharpless asymmetric dihydroxylation (ad) [12] and asymmetric aminohydroxylation (AA) reactions [13]. However, a general and versatile synthetic methodology for obtaining enantiopure chiral 3‐amino‐2‐hydroxy acids is still lacking. In this work, we describe a stereoselective method for the synthesis of chiral 3‐amino‐2‐hydroxy acids and its application to the stereoselective synthesis of natural or pharmaceutically valuable products. Our approach is based on the introduction of the two adjacent stereocenters through a Sharpless ad [ 14] of (*E*)‐α,β‐unsaturated esters **1** followed by a Mitsunobu regioselective and stereoselective azidation on the β hydroxy group [15] to provide, after azido group reduction, the target *erythro* 3‐amino‐2‐hydroxy ester **4**, eventually hydrolyzed to the 3‐amino‐2‐hydroxy acid **5** (Scheme 1). Such procedure has been applied to the synthesis of (2*R*,3*R*)‐3‐phenylisoserine (**5a**), a valuable intermediate for the pharmaceutical industry and epimer of the Taxol side chain, and to (2*R*,3*R*)‐3‐amino‐2‐hydroxy‐6‐methylheptanoic acid (AHMHA) (**5b**), a nonproteinogenic amino acid found in the marine product perthamide C.

## 2 Materials and Methods

### 2.1 General Experimental Procedures

^1^H (400 MHz) and ^13^C (100 MHz) NMR spectra were recorded using a Varian INOVA 400 spectrometer. GC/MS spectra were obtained using an HP 6890 gas chromatograph equipped with an HP‐5975 mass spectrometric detector. HPLC chromatography was carried out with a Shimadzu Nexera LC‐40D quaternary pump equipped with SPD‐M40 PDA detector. Optical rotations were measured at room temperature using a Jasco DIP‐370 polarimeter. Absorption and ECD spectra of compound **2b** were recorded at room temperature using a JASCO J815 spectropolarimeter with a 0.1 mm cell. During the measurements, the instrument was thoroughly purged with nitrogen. Analytical TLC was carried on silica gel 60 Macherey–Nagel sheets. The spots were visualized by exposing the plates to UV radiation (254 nm) and/or by spraying them with a potassium permanganate solution. Column chromatography was performed using silica gel (Merck, Kieselgel 60, 60–230 mesh). THF was freshly distilled before use over sodium benzophenone ketyl under nitrogen atmosphere. Other analytical‐grade solvents and commercially available reagents were used without further purification. Biphenyl dimethyl acetal **8** was synthesized starting from 2,2′‐bridged biphenyl ketone, following the procedure reported in the literature [16].

### 2.2 Synthetic Procedures

#### 2.2.1 (2*R*,3*S*)‐3,4‐Dihydroxy‐4‐phenylpropanoate Ethyl Ester (**2a**)

In a mixture of *tert*‐butanol and water (1:1 *v*/*v*; 90 mL), ad‐mix‐α (11.5 g) and methanesulfonamide (790 mg; 8.3 mmol) were sequentially added. The solution was vigorously stirred for 10 min at room temperature. Subsequently, the mixture was cooled to 0°C using an ice bath, ethyl cinnamate (**1a**) (1.5 g, 1.45 mL, 8.5 mmol) was added dropwise, and the mixture left stirring at 0°C for 19 h. The reaction was then quenched by addition of sodium metabisulfite stirring for 30 min and extracted three times with ethyl acetate. The combined organic phases were washed with brine and dried over anhydrous sodium sulfate. The solvent was evaporated under reduced pressure, and the crude product was purified by silica gel chromatography (*n*‐hexane:ethyl acetate 1:1) yielding **2a** as a white solid with 88% yield. m.p. = 72°C–74°C; [α]^20^_D_ = +4.7 (*c* = 1.00 in ethanol) (Lit [17] for enantiomer (2*S*,3*R*) [α]^20^_D_ = −4.1 [*c* = 1.45 in ethanol]). Daicel Chiralcel OJ column, λ = 220 nm, n‐hexane: *i*‐PrOH = 90:10; flow 0.5 mL/min; ee > 98%, first enantiomer eluted (2*R*,3*S*)‐**2a**. MS (EI): *m*/*z* 210 (M^+^, 1), 119 (12), 107 (68), 104 (100), 91 (30), 79 (64), 76 (85), 51 (14), 31 (5); ^1^H‐NMR (400 MHz, CDCl_3_, δ): 7.29–7.40 (m, 5H), 4.99 (dd, J = 6.9, 3.2 Hz, 1H), 4.34 (dd, J = 5.9, 3.2 Hz, 1H), 4.25 (q, J = 7.2 Hz, 2H), 3.24 (d, J = 6.4 Hz, 1H), 2.92 (d, J = 7.2 Hz, 1H),1.26 (t, J = 7.2 Hz, 3H). ^13^C‐NMR (100 MHz, CDCl_3_, δ): 172.9, 140.1, 128.6, 128.2, 126.4, 74.8, 74.4, 63.3, 14.2.

#### 2.2.2 (2*R*,3*R*)‐3‐Azido‐2‐hydroxy‐3‐phenylpropanoate Ethyl Ester (**3a**)

To a solution of the diol **2a** (100 mg; 0.48 mmol) and triphenylphosphine (164 mg; 0.63 mmol) (both thoroughly dried for 4 h under high vacuum) in 9 mL of anhydrous THF, diisopropyl azodicarboxylate (DIAD) (142 μL; 0.72 mmol) was added at 0°C, cooling with an ice bath. After stirring the reaction mixture for 3 h under an inert atmosphere, trimethylsilyl azide (CH_3_SiN_3_) (130 μL; 0.97 mmol) was added. The resulting solution was stirred for additional 3 h at 0°C and then allowed to warm to room temperature for 48 h. Subsequently, the solvent was removed under reduced pressure, and the residue was dissolved in THF (1.5 mL) and treated with a 1.0 M solution of tetrabutylammonium fluoride in THF (TBAF, 1.3 mL) and water (64 μL). The mixture was stirred at room temperature for 19 h until the disappearance of the intermediate silazide was observed. After evaporating the solvent at reduced pressure, the crude reaction mixture was dissolved in the minimal amount of dichloromethane and purified by silica gel chromatography (petroleum ether:ethyl acetate 1:1). The product **3a** was obtained as a colorless oil in 40% yield. [α]^20^_D_ = −43.3 (*c* = 1.00 in ethanol). ^1^H‐NMR (40 0 MHz, CDCl_3_, δ): 7.32–7.37 (m, 5H), 4.87 (d, J = 3.6 Hz, 1H), 4.51 (d, J = 3.2 Hz, 1H), 4.16 (q, J = 7.2 Hz, 2H), 2.91 (bs, 1H), 1.18 (t, J = 7.2 Hz, 3H). ^13^C‐NMR (100 MHz, CDCl_3_, δ): 171.3, 134.4, 128.9, 128.6, 127.8, 73.7, 67.2, 62.2, 14.0.

#### 2.2.3 (2*R*,3*R*)‐3‐Amino‐2‐hydroxy‐3‐phenylpropanoate Ethyl Ester (**4a**)

The ester **3a** (33 mg, 0.14 mmol) was dissolved in anhydrous ethanol (2 mL), and then a suspension of 10% palladium on carbon (Pd/C, 20 mg) was added for approximately 24 h under a hydrogen atmosphere. The reaction was stopped by filtering the mixture through a celite plug and washing it with ethanol. Excess solvent was removed by evaporation under reduced pressure, yielding product **4a** as a colorless oil with 79% yield. [α]^20^_D_ = −24.5 (*c* = 0.98 in ethanol). ^1^H‐NMR (400 MHz, CDCl_3_, δ): 7.26–7.35 (m, 5H), 4.46 (d, J = 3.9 Hz, 1H), 4.32 (d, J = 3.2 Hz, 1H), 4.09 (q, J = 7.0, 2H), 2.61 (bs, 3H), 1.17 (t, J = 7.1, 3H). ^13^C‐NMR (100 MHz, CDCl_3_, δ): 172.5, 140.3, 128.3, 127.8, 127.0, 74.7, 61.5, 58.2, 14.0.

#### 2.2.4 (2*R*,3*R*)‐3‐Amino‐2‐hydroxy‐3‐phenylpropanoic Acid (**5a**)

The amino hydroxy ester **4a** (0.11 mmol) was dissolved in THF (2 mL), and 150 μL of a 2.0 M aqueous LiOH solution was added. The reaction mixture was heated at reflux overnight. After completion, the solvent was removed under reduced pressure and the residue was dissolved in distilled water (2 mL). The resulting solution was neutralized by the addition of sodium dihydrogen phosphate dihydrate (NaH_2_PO_4_·2H_2_O, as needed) and extracted three times with ethyl acetate. The aqueous layer, containing the desired amino acid, was concentrated, and the resulting solid was treated with methanol, filtered, and dried under reduced pressure to afford compound **5a** as a white solid in 60% yield. [α]^20^_D_ = +22.3 (*c* = 1.00 in methanol) [Lit [18]. [α]^20^_D_ = +3.6 (*c* = 0.5 in 6 M HCl)]. ^1^H‐NMR (400 MHz, CD_3_OD, δ): 7.46–7.48 (m, 2H), 7.34–7.36 (m, 2H), 4.54 (d, J = 4.4 Hz, 1H), 4.35 (d, J = 4.4 Hz, 1H), 3.29 (s, 2H). ^13^C‐NMR (100 MHz, CD_3_OD, δ): 174.4, 133.6, 128.6, 128.2, 128.1, 71.7, 58.6.

#### 2.2.5 4‐Methylpentanal (**7**)

Pyridinium chlorochromate (PCC, Corey's reagent) (6.34 g; 29.4 mmol; 2.0 eq), sodium acetate (241.0 mg; 2.94 mmol; 0.2 eq), and neutral aluminum oxide (Al_2_O_3_) (3.2 g) were mixed in anhydrous dichloromethane (60 mL) under a nitrogen atmosphere. The solution was cooled to 0°C using an ice bath, and 4‐methylpentanol (**6**) (1.82 mL; 1.5 g; 14.7 mmol) was added. The resulting reaction mixture was stirred at room temperature for 1.5 h. The brown colored solution was filtered through a silica gel column using only dichloromethane as the eluent. The separated product, highly volatile, was concentrated at 25°C–30°C under controlled pressure (800 mmHg), resulting in a colorless oil obtained with quantitative yield. MS (EI): *m*/*z* 100 (M+, 1), 72 (15), 57 (100), 56 (92), 43 (56), 41 (60). ^1^H‐NMR (400 MHz, CDCl_3_, δ): 9.77 (s, 1H), 2.42 (t, J = 7.6 Hz, 2H), 1.49–1.63 (m, 3H), 0.91 (d, J = 6.4 Hz, 6H). ^13^C‐NMR (100 MHz, CDCl_3_, δ): 180.0, 33.6, 32.2, 27.2, 22.4, 23.3.

#### 2.2.6 Ethyl 6‐Methylhept‐2‐enoate (**1b**)

The aldehyde (**6**) and triethyl phosphonoacetate (TEPA) (3.20 mL; 1.1 eq) were added to a solution of LiOH (387 mg; 1.1 eq) in anhydrous THF (16.0 mL) under a nitrogen atmosphere. The resulting mixture was stirred at room temperature for 24 h. Once the reaction was deemed complete, it was quenched with distilled water (10 mL). The mixture was extracted three times with ethyl acetate. The combined organic phases were dried over sodium sulfate (Na_2_SO_4_) and concentrated at 40°C under reduced pressure (below 150 mmHg). The ester was purified using a silica gel chromatography column with a mixture of petroleum ether/ethyl acetate (96:4), resulting in the product with a quantitative yield. MS (EI): *m*/*z* 170 (M+, 1), 125 (64), 115 (100), 101 (89), 73 (65), 55 (78), 41 (37); ^1^H‐NMR (400 MHz, CDCl_3_, δ): 6.97 (dt, J = 15.6, 7.2 Hz, 1H), 5.81 (d, J = 15.6 Hz, 1H), 4.18 (q, J = 7.6 Hz, 2H), 2.20 (q, J = 7.2 Hz, 2H), 1.52–160 (m, 1H), 1.34 (q, J = 7.6 Hz, 2H), 1.29 (t, J = 7.6 Hz, 3H), 0.89 (d, J = 6.8 Hz, 6H). ^13^C‐NMR (100 MHz, CDCl_3_, δ): 166.7, 149.6, 121.0, 60.0, 37.0, 30.0, 27.4, 22.3, 14.2.

#### 2.2.7 (2*R*,3*S*)‐2,3‐Dihydroxy‐6‐methylheptanoate Ethyl Ester (**2b**)

In a mixture of *tert*‐butanol and water (1:1 v/v; 164 mL), ad‐mix‐α (21.6 g; 3.12 mmol) and methanesulfonamide (MeSO_2_NH_2_) (1.48 g; 0.22 mmol) were sequentially added. The solution was vigorously stirred for 10 min at room temperature. Subsequently, the mixture was cooled to 0°C using an ice bath, and the olefinic ethyl ester **1b** (2.805 g; 16 mmol) was added dropwise. After 19 h, the reaction was quenched by adding equimolar sodium metabisulfite (Na_2_S_2_O_5_) to the mixture and stirring for about 30 min. At the end of this time, the reaction mixture was extracted three times with ethyl acetate. The combined organic phases were washed with brine and then dried over sodium sulfate. The solvent was evaporated under reduced pressure, and the crude product was purified by silica gel chromatography (petroleum ether: ethyl acetate 4:1). The pure diol appears as a colorless oil, obtained with 68% yield. [α]^20^_D_ = −13.1 (*c* = 1.07 in ethanol); MS (EI): *m*/*z* 104 (100), 83 (11), 76 (76), 55 (13), 43 (13) 41 (15). ^1^H‐NMR (400 MHz, CDCl_3_, δ): 4.29 (q, J = 7.2 Hz, 2H), 4.08 (dd, J = 5.2, 2.0 Hz, 1H), 3.82–3.88 (m, 1H), 3.11 (d, J = 5.2 Hz, 1H), 1.96 (bd, J = 8.8 Hz, 1H), 1.52–1.64 (m, 3H), 1.35–1.42 (m, 1H), 1.31 (t, J = 7.2 Hz, 3H), 1.19–1.29 (m, 1H), 0.91 (d, J = 6.8 Hz, 6H). ^13^C‐NMR (100 MHz, CDCl_3_, δ): 173.8, 73.1, 72.9, 62.1, 34.8, 31.6, 28.0, 22.6, 22.5, 14.1.

#### 2.2.8 (2*R*,3*R*)‐3‐Azido‐2‐hydroxy‐6‐methylheptanoate Ethyl Ester (**3b**)

To a solution of the diol (200 mg; 0.98 mmol) and triphenylphosphine (Ph_3_P) (340 mg; 1.27 mmol) (both thoroughly dried for 4 h under high vacuum) in 18 mL of anhydrous THF, diisopropyl azodicarboxylate (DIAD) (295 μL; 1.5 mmol) was added at 0°C, cooling with an ice bath. After stirring the reaction mixture for 3 h under an inert atmosphere, trimethylsilyl azide (CH_3_SiN_3_) (318 μL; 1.95 mmol) was added. The resulting solution was stirred for additional 3 h at 0°C and then allowed to warm to room temperature for 5 days. Subsequently, the solvent was evaporated under reduced pressure, and the residue was dissolved in THF (3 mL) and treated with tetrabutylammonium fluoride (TBAF) (2.6 mL) and water (134 μL). The mixture was stirred at room temperature overnight until the disappearance of the intermediate silazide was observed. After evaporating the solvent at reduced pressure, the crude reaction mixture was dissolved in the minimal amount of dichloromethane (CH_2_Cl_2_) and purified by silica gel chromatography (petroleum ether: ethyl acetate 4:1). The pure product appears as an oily and colorless material and was obtained with 77% yield. [α]^20^_D_ = +31.3 (*c* = 1.04 in ethanol); MS (EI): *m*/*z* 104 (22), 76 (59), 72 (20), 56 (31), 43 (100), 41 (35). ^1^H‐NMR (400 MHz, CDCl_3_, δ): 4.24–4.33 (m, 3H), 3.50 (dt, J = 10.4, 3.6 Hz, 1H), 3.18 (d, J = 5.6, 1H), 1.64–1.74 (m, 1H), 1.44–1.60 (m, 2H), 1.35–1.41 (m, 1H), 1.31 (t, J = 6.8 Hz, 1H), 1.21–1.29 (m, 1H), 0.90 (d, J = 6.0 Hz, 3H), 0.89 (d, J = 6.0 Hz, 3H). ^13^C‐NMR (100 MHz, CDCl_3_, δ): 172.2, 73.4, 64.9, 62.4, 35.4, 27.8, 27.0, 22.6, 22.2, 14.1.

#### 2.2.9 (2*R*,3*R*)‐3‐Amino‐2‐hydroxy‐6‐methylheptanoate Ethyl Ester (**4b**)

The ester **3b** (0.44 mmol) was dissolved in anhydrous ethanol, and then a suspension of 10% palladium on carbon (Pd/C) was added for approximately 24 h under a hydrogen atmosphere. The reaction was stopped by filtering the mixture through a celite plug and washing it with ethanol. Excess solvent was removed by evaporation under reduced pressure, yielding the desired product as a colorless oil with a 90% yield. [α]^20^_D_ = +24.4 (*c* = 1.13 in ethanol); ^1^H‐NMR (400 MHz, CDCl_3_, δ): 4.23–4.28 (m, 2H), 4.12–4.18 (m, 1H), 3.00 (m, 1H), 2.21 (bs, 3H), 1.46–1.57 (m, 1H), 1.33–1.38 (m, 2H), 1.30 (t, J = 6.4 Hz, 3H), 1.10–1.22 (m, 2H), 0.88 (d, J = 6.4 Hz, 3H), 0.86 (d, J = 6.4 Hz, 3H). ^13^C‐NMR (100 MHz, CDCl_3_, δ): 173.3, 74.2, 61.4, 54.6, 35.5, 30.1, 28.0, 22.6, 22.3, 14.2.

#### 2.2.10 (2*R*,3*R*)‐3‐Amino‐2‐hydroxy‐6‐methylheptanoic acid (AHMHA) (**5b**)

The amino hydroxy ester **4b** (0.25 mmol) was dissolved in THF (3 mL), and 400 μL of a 2.0 M aqueous LiOH solution were added. The reaction mixture was heated at reflux for 3 h. After completion, the solvent was removed under reduced pressure and the residue was dissolved in distilled water (7 mL). The resulting solution was neutralized by the addition of sodium dihydrogen phosphate dihydrate (NaH_2_PO_4_·2H_2_O, as needed) and extracted three times with ethyl acetate. The aqueous layer, containing the desired amino acid, was concentrated, and the resulting solid was treated with methanol, filtered, and dried under reduced pressure to afford AHMHA as a white solid in 62% yield. [α]^20^_D_ **=** +14.5 (*c* = 1.08 in methanol); ^1^H‐NMR (400 MHz, CD_3_OD, δ): 4.10 (bd, J = 3.6 Hz,1H), 3.37–3.42 (m, 1H), 1.51–1.65 (m, 3H), 1.23–1.34 (m, 2H), 0.90 (d, J = 6.4 Hz, 3H), 0.89 (d, J = 6.4 Hz, 3H). ^13^C‐NMR (100 MHz, CD_3_OD, δ): 176.6, 72.2, 55.8, 35.6, 29.2, 27.0, 22.9, 22.5.

#### 2.2.11 Biphenyl Boronate **2c**

Under an inert atmosphere, diol **2b** (0.36 mmol) was dissolved in anhydrous chloroform (5 mL). Subsequently, 4‐biphenylboronic acid (0.44 mmol) and activated 4 Å molecular sieves were added, and the mixture was stirred at room temperature overnight. The mixture was then filtered to remove the molecular sieves, and the solvent was evaporated. The resulting crude product was purified by column chromatography, eluting with chloroform alone, affording the pure boronate **2c** as a colorless liquid in 90% yield. (*R*,*R*)‐Whelk‐O1 and (*S*,*S*)‐Whelk‐O1 columns (250 mm × 4.6 mm, 5 μm); λ = 260 nm; hexane:*i*‐PrOH = 99:1 (v/v); flow 0.8 mL/min; ee 97.5%. The (2*R*,3*S*)‐**2c** enantiomer eluted first using (*R*,*R*)‐Whelk‐O1 and second using (*S*,*S*)‐Whelk‐O1. ^1^H‐NMR (400 MHz, CDCl_3_, δ): 7.97 (d, J = 8.00 Hz, 2H), 7.67 (d, J = 4.8 Hz, 2H), 7.65 (d, J = 4.2 Hz, 2H), 7.47 (t, J = 7.4 Hz, 2H), 7.38 (t, J = 7.4 Hz, 1H), 4.63 (d, J = 6.2 Hz, 1H), 4.55 (q, J = 6.2 Hz, 1H), 4.30 (q, J = 7.0, 2H), 1.82 (q, J = 7.5 Hz, 6H), 1.61–1.71 (m, 1H), 1.44–1.53 (m, 1H), 1.34 (t, J = 7.0 Hz, 3H), 1.30–1.42 (m, 1H), 0.96 (d, J = 6.4 Hz, 6H). ^13^C‐NMR (100 MHz, CDCl_3_, δ): 171.0, 144.3, 140.8, 135.6, 128.8, 127.7, 127.3, 126.6, 81.3, 80.0, 61.6, 34.5, 33.6, 27.8, 22.6, 22.5, 14.2.

#### 2.2.12 Biphenyldioxolane **2d**

The biphenyl acetal **8** (100 mg, 0.40 mmol) was dissolved in anhydrous CHCl_3_ (5 mL) under a nitrogen atmosphere. Subsequently, the diol **2b** (0.40 mmol, 1equiv.) was added to the mixture along with traces of *p*‐toluenesulfonic acid and previously activated 4 Å molecular sieves. The mixture was stirred at room temperature for 24 h. After filtration, evaporation of the solvent, and column chromatography the dioxolanes **2d** was obtained in 45% yield. Its CD and UV spectra were recorded in ACN between 190 and 330 nm. MS (EI): *m*/*z* 394.2 (M^+^,91), 208.1 (22), 180.1 (65), 179.1 (100), 178.1 (47), 166.0 (13), 165.0 (60), 95.1 (10). ^1^H‐NMR (400 MHz, CDCl_3_, δ): 7.41 (t, J = 7.0 Hz, 2H), 7.32 (t, J = 7.3 Hz, 2H), 7.31 (d, J = 4.0 Hz, 4H), 4.25 (m, 7H), 3.57 (m, 1H), 1.80 (m, 3H), 1.35 (m, 5H), 0.92 (d, J = 6.5 Hz, 6H). ^13^C‐NMR (100 MHz, CDCl_3_, δ): 167.1, 140.2, 136.2, 129.3, 127.3, 122.8, 79.8, 75.6, 59.7, 44.9, 36.6, 27.8, 25.6, 23.1, 14.1.

## 3 Results and Discussion

### 3.1 Synthesis of 3‐Amino‐2‐hydroxy Acids

At first, the approach was tested on the synthesis of optically active 3‐phenylisoserine **5a** (Scheme 1), submitting ethyl cinnamate (**1a**) to ad with ad‐mix‐α reagent and methanesulfonamide in a 1:1 tert‐butanol/water mixture, providing the diol (2*R*,3*S*)‐diol **2a** in 80% yield and 98% ee after column chromatography. Diol **2a** was then submitted to a Mitsunobu reaction with trimethylsilylazide (Me_3_SiN_3_) as a nucleophile [19] to yield *erythro* (2*R*,3*R*) azide **3a**. Notably, the Mitsunobu reaction was completely regioselective and stereoselective, allowing the substitution of only the β‐hydroxy group with the amino functionality having inverted configuration and thus allowing achieving the desired 3‐amino‐2‐hydroxy ester with the wanted relative and absolute configuration. The regioselective and stereoselective Mitsunobu substitution of *syn*‐2,3‐dihydroxy esters with hydrazoic acid has been previously described by Ko [15] and applied to the synthesis of statine. This author postulated that in 2,3‐dihydroxy esters the electron‐withdrawing effect of the ester moiety makes the α‐hydroxyl group more acidic and less nucleophilic than the β‐hydroxyl. Therefore, in non‐basic Mitsunobu conditions where hydroxyls are not deprotonated, the more nucleophilic β‐hydroxyl group reacts with the electrophilic phosphonium adduct of DIad (or DEAD), eventually giving rise to nucleophilic substitution. The (2*R*,3*R*)‐amino hydroxy ester **4a** was then obtained by reducing (2*R*,3*R*)‐azide **3a** in the presence of hydrogen and palladium on carbon [20]. The subsequent hydrolysis of **4a** with LiOH in a THF/water mixture provided (2*R*,3*R*)‐3‐phenylisoserine **5a** in 20% overall yield. Similarly, the enantiomers of **2a**‐**5a** could be obtained by the use of AD‐mix‐β reagent mixture in the AD step.

Subsequently, this approach was applied to the synthesis of the natural product 3‐amino‐2‐hydroxy‐6‐methylheptanoic acid (AHMHA, **5b**), a component of the cyclic octapeptide perthamide C recovered from the marine sponge *Theonella swinhoei* [21]. The relative and absolute configuration of AHMHA (**5b**) was established by the group of Zampella and coworkers [22] through enantioselective total synthesis of both *erythro*‐(2*R*,3*R*) and *threo*‐(2*R*,3*S*) diastereomers. Comparison of ^1^H and ^13^C NMR spectra of natural and synthetic samples allowed us to assign *erythro* configuration to the two stereogenic centers of natural AHMHA while its (2*R*,3*R*) absolute configuration was determined using the Marfey method [23] and confirmed by optical rotation values comparison.

**SCHEME 2:**
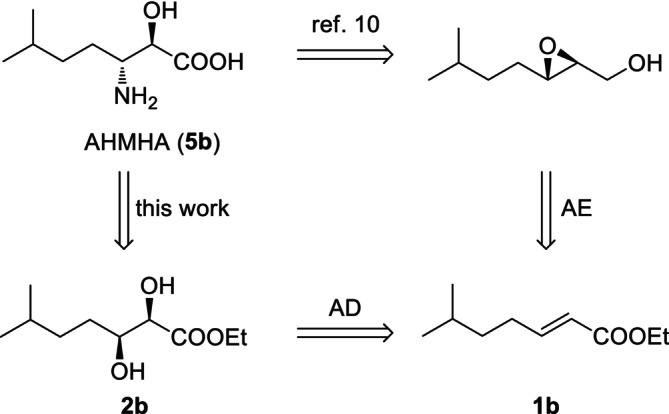
Retrosynthetic analyses of (2*R*,3*R*)‐3‐amino‐2‐hydroxy‐6‐methylheptanoic acid (AHMHA, **5b**).

The AHMHA synthetic procedure reported in literature involves seven consecutive reactions, with the enantioselective step featuring a Sharpless asymmetric epoxidation (AE) reaction [24]. This approach is based on the reduction of α,β‐unsaturated ester **1b** to an allylic alcohol, then submitted to AE (Scheme 2). Furthermore, the obtained epoxy alcohol is re‐oxidized to a chiral epoxy acid, subsequently regioselectively opened with sodium azide, and reduced to the desired 3‐amino‐2‐hydroxy acid AHMHA. Such synthetic procedure requires first the reduction of the carboxy moiety to alcohol and then a reoxidation of the hydroxy group back to the carboxylic acid, thus making the pathway lengthy.

Therefore, we found it worthwhile to apply to the synthesis of AHMHA (**5b**) our more direct and efficient approach. In fact, by introducing the two adjacent stereocenters through an ad reaction on ester **1b**, it allows us to avoid the reduction and oxidation steps required in Zampella's synthesis (Scheme 2).

**SCHEME 3:**
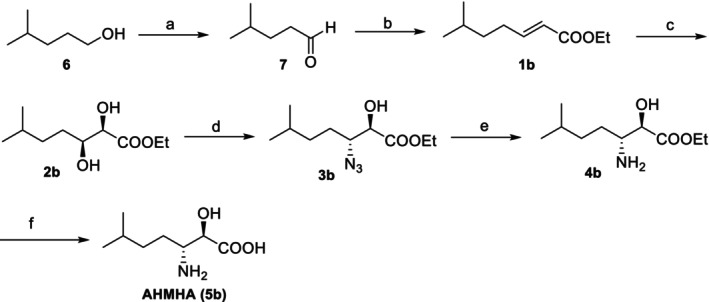
Synthetic sequence for AHMHA. Reagents and conditions: (a) PCC, CH_3_COONa, neutral Al_2_O_3_, CH_2_Cl_2_; (b) LiOH, TEPA, THF rt; (c) ad‐mix‐α, *t‐*BuOH/H_2_O, MeSO_4_NH_2_, 0°C; (d) Me_3_SiN_3_, PPh_3_, DIad, THF, 0°C to rt; (e) H_2_, Pd/C, EtOH, rt; (f) LiOH 2 M, reflux THF, then NaH_2_PO_4_·2H_2_O.

Accordingly, commercially available 4‐methylpentanol (**6**) was oxidized to aldehyde **7** in quantitative yield by pyridinium chlorochromate (PCC) treatment (Scheme 3). Subsequently, aldehyde **7** was subjected to a Horner–Wadsworth–Emmons (HWE) olefination reaction [25] with triethyl phosphonoacetate (TEPA) in the presence of LiOH, providing the α,β‐unsaturated ethyl ester **1b** in quantitative yield after chromatographic purification. Sharpless AD of ester **1b** using the AD‐mix‐α reagent and methanesulfonamide in a 1:1 tert‐butanol/water mixture provided (2*R*,3*S*)‐diol **2b** in 68% yield and high enantiopurity (*vide infra*) after chromatographic purification. The absolute configuration of diol **2b** was preliminarily assigned by the Sharpless' mnemonic rule [14] and then confirmed by electronic circular dichroism (ECD) analysis with a biphenyl chiroptical probe (see the following paragraph). Diol **2b** was then subjected to a Mitsunobu reaction with Me_3_SiN_3_ as a nucleophile [19] to yield (2*R*,3*R*) azide **3b**. Notably, the latter reaction was again completely regioselective and stereoselective, thus allowing the achievement of the *erythro* ester **4b** with the correct relative and absolute configuration. The (2*R*,3*R*)‐amino hydroxy ester **4b** was obtained by hydrogenation of (2*R*,3*R*)‐azide **3b** in the presence of hydrogen and palladium on carbon [20]. Finally, (2*R*,3*R*)‐**5b** was obtained via a basic hydrolysis [26] of (2*R*,3*R*)‐**4b** with a 2.0 M aqueous solution of LiOH in THF under reflux, followed by treatment with NaH_2_PO_4_^**·**^2H_2_O until neutrality. Optical rotation of the synthetic (2*R*,3*R*)‐AHMHA was [α]^20^_D_ = +14.5 (*c* = 1.08 in methanol), in agreement in sign and order of magnitude with that of the natural compound having [α]^20^_D_ = +8.8 (*c* = 0.55 in methanol) [22] and thus allowing it to confirm its (2*R*,3*R*) absolute configuration.

### 3.2 Stereochemical Analysis of Diol **2b**

**FIGURE 1:**
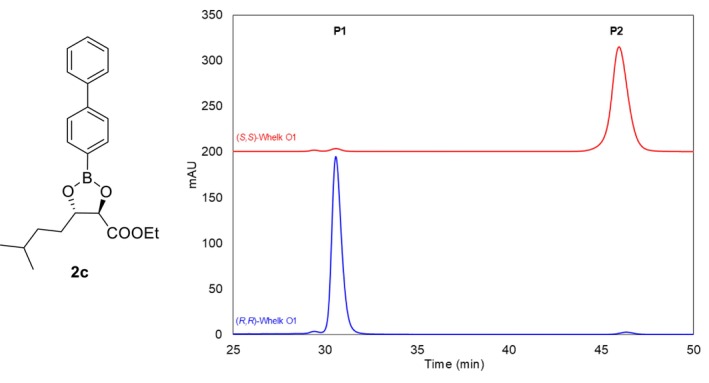
HPLC separation of **2c**. Blue traces: (*R*,*R*)‐Whelk‐O1, 250 mm × 4.6 mm (5 μm); red traces: (*S*,*S*)‐Whelk‐O1, 250 mm × 4.6 mm (5 μm). Mobile phase: hexane:isopropanol (99:1, *v*/*v*). Flow rate: 0.8 mL/min. UV detection at 260 nm. P1 = 30.57 min, P2 = 45.96 min. The blue chromatogram was shifted by +1.58 min for better comparison.

To determine the enantiopurity of diol **2b** by HPLC on a chiral stationary phase (CSP), it was first necessary to insert a chromophoric moiety on the molecule to allow its UV detection. Therefore, **2b** was converted in the corresponding 4‐biphenylboronate **2c** by reaction with 4‐biphenyl boronic acid in CHCl_3_ in the presence of activated 4 Å molecular sieves [27, 28]. Having only one enantiomer of **2c** in hand and to overcome the necessity of a racemate to identify the eluted enantiomers peaks, we resorted to the so‐called “inverted chirality columns approach” (ICCA), which consists of the use of CSPs available in both enantiomeric forms [29]. In this case, inversion of the elution order for a pair of enantiomers is observed in response to the change in column chirality, thus generating a virtual racemate. Accordingly, **2c** was eluted on enantiomeric CSP (*S*,*S*)‐Whelk O1 and (*R*,*R*)‐Whelk O1, observing inverted peaks retention time on the two columns for the same sample and determining a 97.5% ee of boronate **2c** and then of chiral diol **2b** (Figure 1).

**SCHEME 4:**
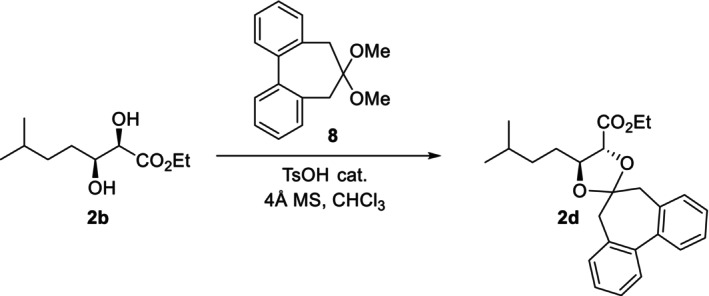
Synthesis of biphenyldioxolane **2d**.

Due to the empirical nature and the potential for misassignments of the Sharpless mnemonic rule [30], the absolute configuration of diol **2b** was independently assigned by the use of flexible biphenyl chiroptical probes. Such an approach has been extensively developed by our research group for determining the absolute configuration of flexible and non‐chromophoric compounds and applied to diols [16, 31], amines [32], and carboxylic acids [33, 34, 35], including the application to fairly complex natural compounds [36]. This method proved to be particularly direct, simple, and general for determining the absolute configuration of non‐chromophoric aliphatic chiral cyclic and acyclic 1,2‐, 1,3‐, and 1,4‐diols [16, 31]. Accordingly, diols are converted into their corresponding biphenyl dioxolanes (Scheme 4), resulting in a pair of diastereomers, each characterized by either a *P* or an *M* biphenyl twist.

**FIGURE 2:**
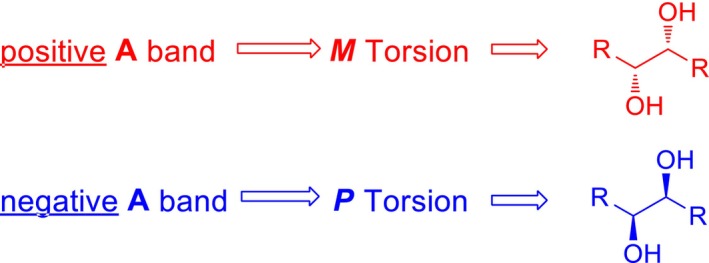
Nonempirical rule scheme relating the absolute configuration of *threo* diols with the sign of the A band (at ~250 nm) in the ECD spectrum of their biphenyl dioxolanes.

At room temperature, the low rotational barrier of biphenyl (approximately 14 kcal/mol) allows thermodynamic equilibrium between the two diastereomers, with the more stable of the two being the most populated. The biphenyl twist can be derived by the sign of a diagnostic Cotton effect at 250 nm (biphenyl A‐band) in the ECD spectrum. A positive Cotton band corresponds to a *M* twist, whereas a *P* twist is associated with a negative band in the spectrum. After elucidating the mechanism of chirality induction from the chiral diol to the biphenyl probe, a simple nonempirical rule was established to relate the absolute configuration of the diol to the sign of the diagnostic ECD A band at 250 nm (Figure 2) [37].

Such approach was applied to **2b**, converting it into its corresponding dioxolane **2d** by reaction with dimethyl acetal **8** in chloroform in the presence of trace amounts of *p*‐toluenesulfonic acid and 4 Å molecular sieves (Scheme 4). The product **2d** was isolated after filtration, solvent evaporation, and purification by column chromatography.

**FIGURE 3:**
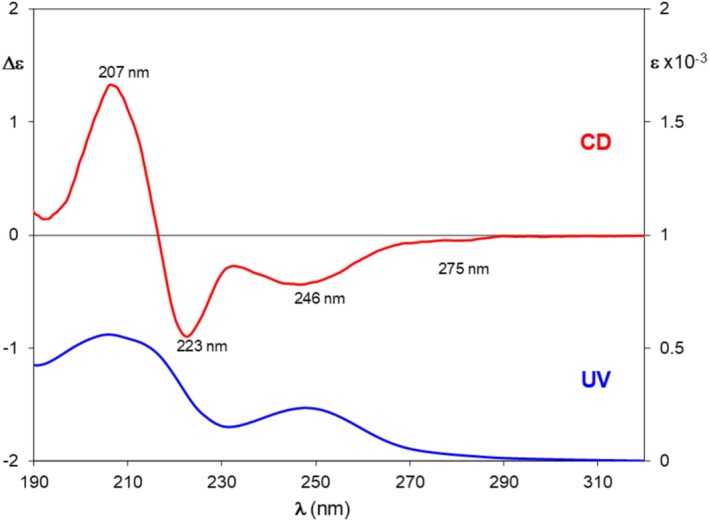
Experimental UV (blue line) and ECD (red line) spectra of biphenyldioxolane **2d**, recorded in ACN.

The ECD and UV spectra of compound **2d** (Figure 3) were recorded in the 190‐ to 320‐nm range in acetonitrile. The ECD spectrum shows the typical spectral shape observed in diol biphenyl dioxolanes displaying a high amplitude band with a negative Cotton effect occurring at 246 nm (i.e., in correspondence to the A band), followed by a negative and positive Cotton effects at 223 and 207 nm, respectively.

As reported in Figure 2, a negative Cotton effect of the diagnostic A band is associated with a *P* twist of the aryl‐aryl bond in the biphenyl. In the case of *threo* diols such as **2b**, this correlation leads to the assignment of the absolute configuration (2*R*,3*S*) for the C‐2 and C‐3 stereocenters [28], confirming the empirical assignment carried out by the Sharpless rule.

## 4 Conclusions

We described herein a straightforward enantioselective synthetic strategy to obtain chiral *erythro* 3‐amino‐2‐hydroxy acids from aromatic and aliphatic α,β‐unsaturated esters and we applied it to the synthesis of optically active 3‐phenyl isoserine (**5a**) and AHMHA (**5b**), a component of perthamide C, an oligopeptide isolated from the Pacific sponge *Theonella swinhoei* known for its anti‐inflammatory and antipsoriatic properties. Both aminohydroxy acids were obtained in high enantiopurity (~98% ee) and 20%–30% overall yield. In particular, aminohydroxy acid (2*R*,3*R*)‐AHMHA (**5b**) was obtained with very high enantiopurity and in 30% overall yield in just six synthetic steps, starting from commercially available 4‐methylpentanol. The synthetic methodology we proposed has proven to be more efficient and direct compared to the one previously reported in the literature. While the previous procedure introduced the two stereocenters of the molecule through AE followed by regioselective opening of the oxirane ring, in this procedure we introduced the two stereocenters through Sharpless ad and we achieved the regioselective and stereospecific amination at the β‐position by Mitsunobu reaction on the diol. The absolute configuration of diol **2b**, precursor of (2*R,*3*R*)‐AHMHA (**5b**), was assigned by ECD analysis of its biphenyl dioxolane, thereby also confirming the absolute configuration of the natural AHMHA.

## Supporting information


**Data S1:** Supporting information.

## Data Availability

The data that support the findings of this study are available in the supporting information of this article.
