# Supplementary material for: Practical Enantioselective Approach to 3‐Amino‐2‐Hydroxy Acids and Application to the Synthesis of Natural Products
Source: Chirality. 2026 Jun 17;38(7):e70115. doi: 10.1002/chir.70115 (PMC13276025; doi:10.1002/chir.70115)
Supplement: Supplementary file 1 — Data S1: Supporting information. [file CHIR-38-e70115-s001.pdf]

Supporting Information

Practical enantioselective approach to 3-amino-2-hydroxy acids and application to the synthesis of natural products

Marilena Caporale, Giulia Marsico, Ernesto Santoro, Patrizia Scafato, and Stefano Superchi*

Department of Basic and Applied Sciences, University of Basilicata, Viale dell'Ateneo Lucano 10, 85100 Potenza, Italy.

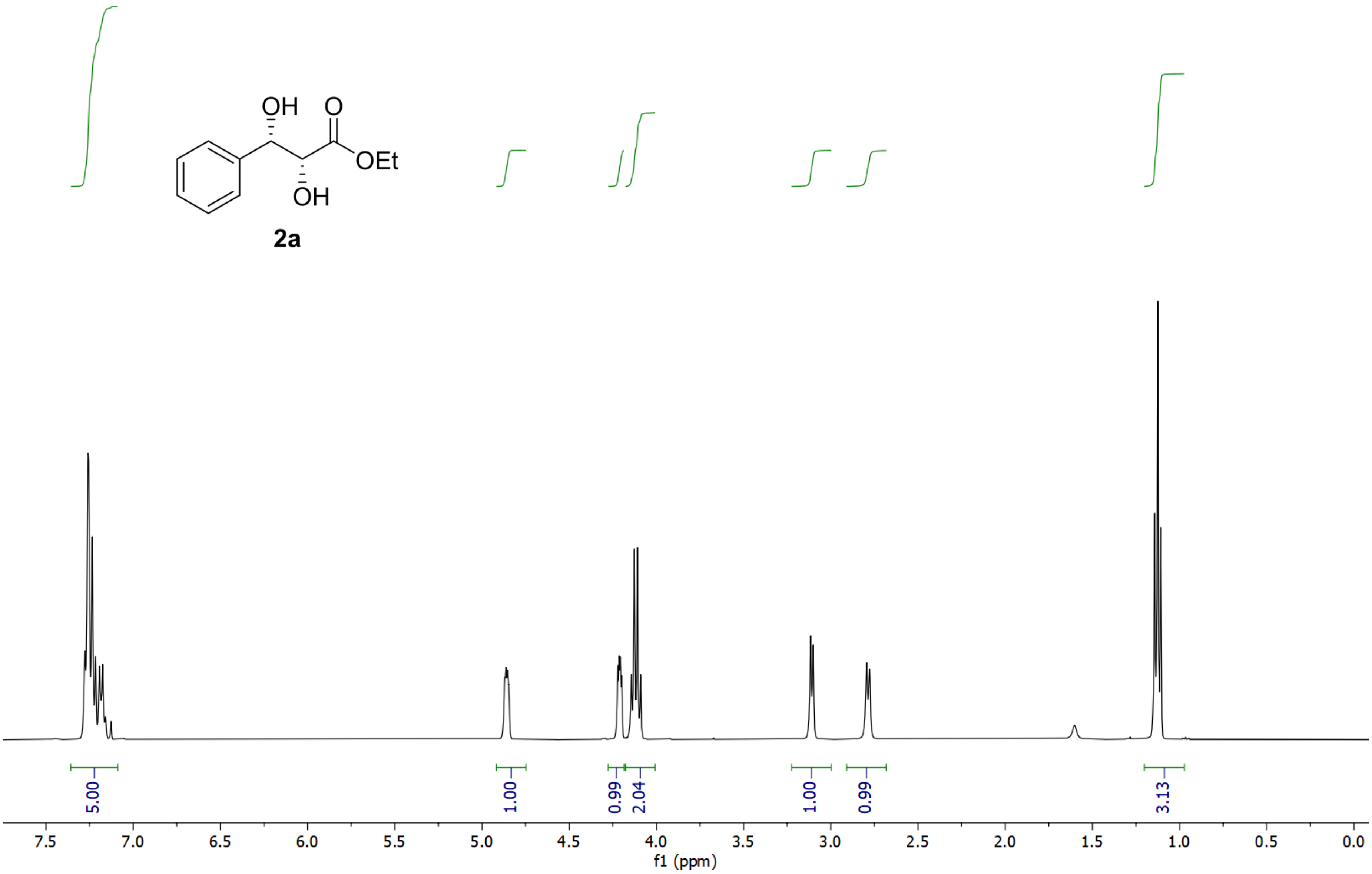
OH
O
OEt
OH
2a
5.00
1.00
0.99
2.04
1.00
0.99
3.13
7.5
7.0
6.5
6.0
5.5
5.0
4.5
4.0
3.5
3.0
2.5
2.0
1.5
1.0
0.5
0.0
f1 (ppm)

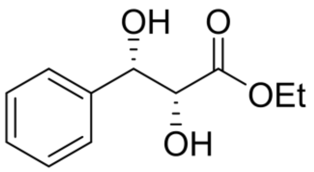
OH
O
OEt
OH

2a

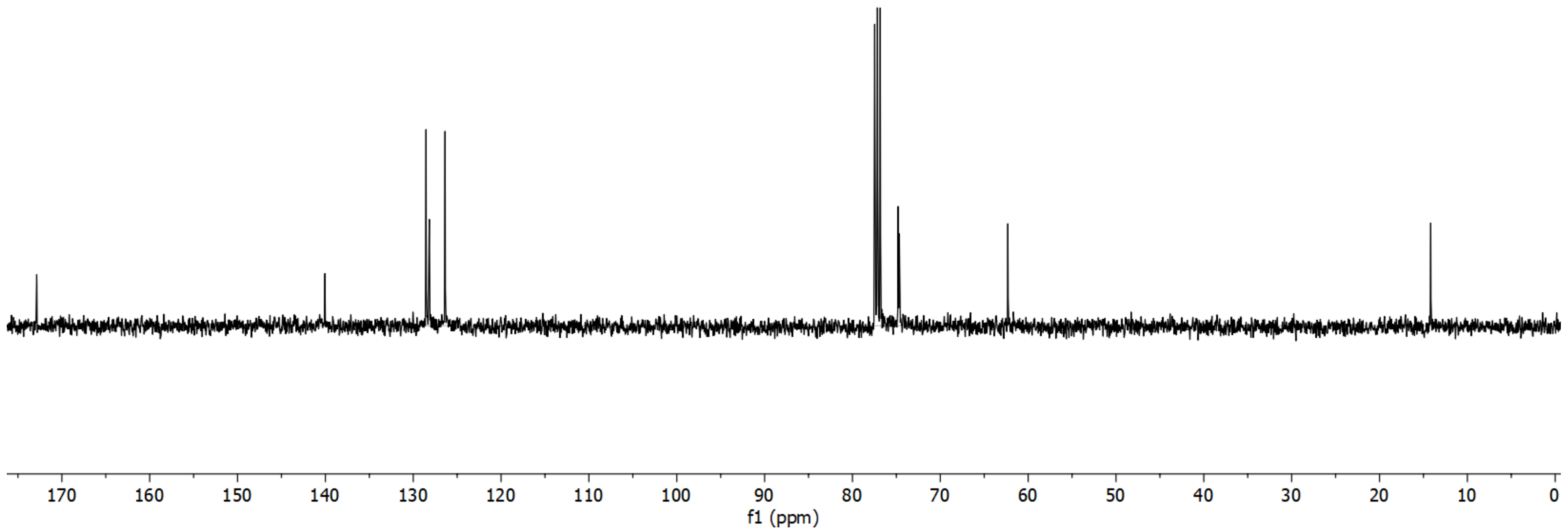
170
160
150
140
130
120
110
100
90
80
70
60
50
40
30
20
10
0
f1 (ppm)

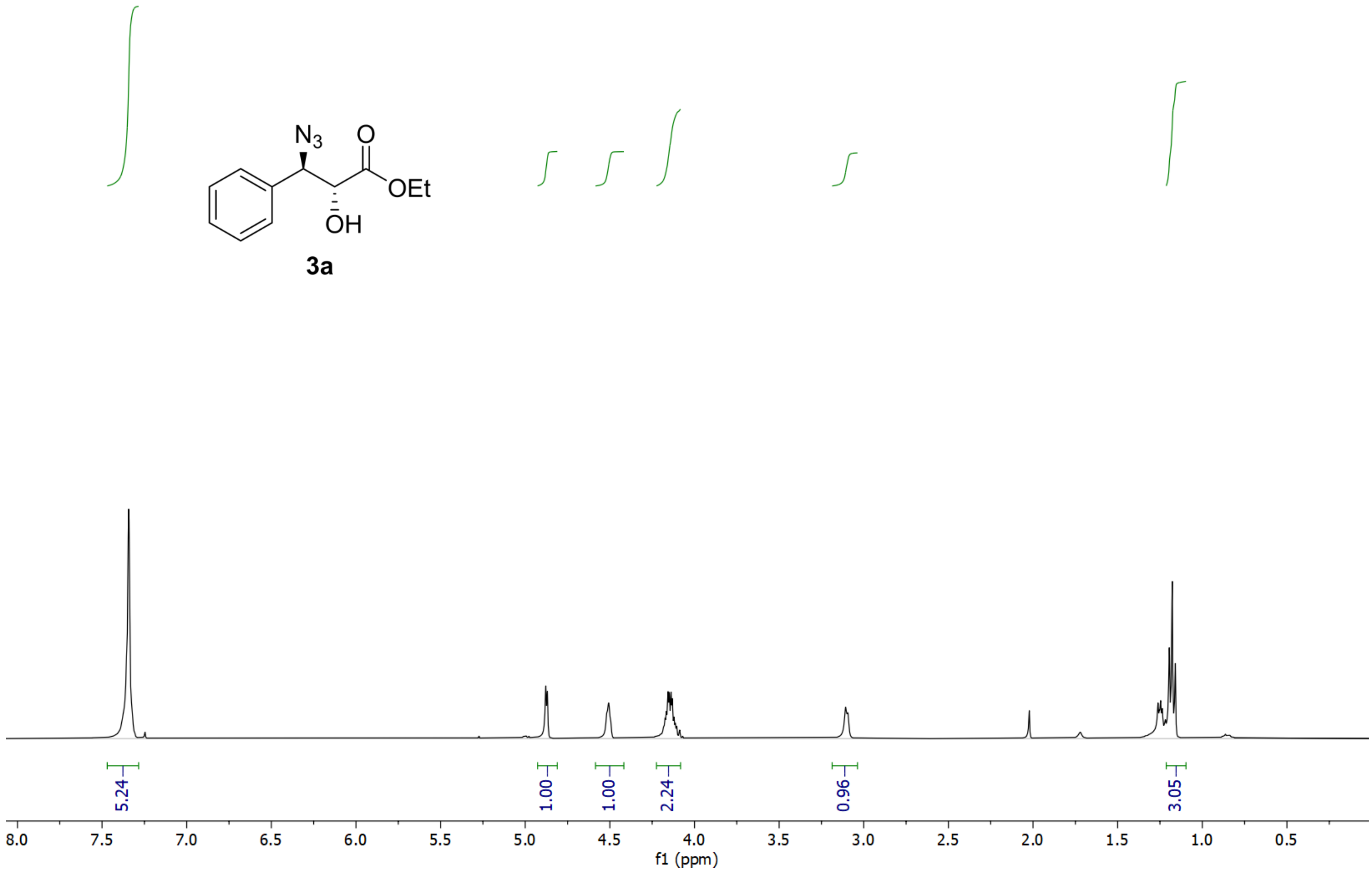
N3
O
OEt
OH
5.24
1.00
1.00
2.24
0.96
3.05
8.0
7.5
7.0
6.5
6.0
5.5
5.0
4.5
4.0
3.5
3.0
2.5
2.0
1.5
1.0
0.5
f1 (ppm)

3a

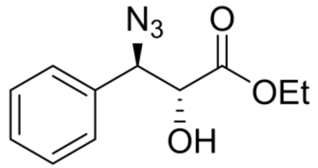
N3
O
OEt
OH

3a

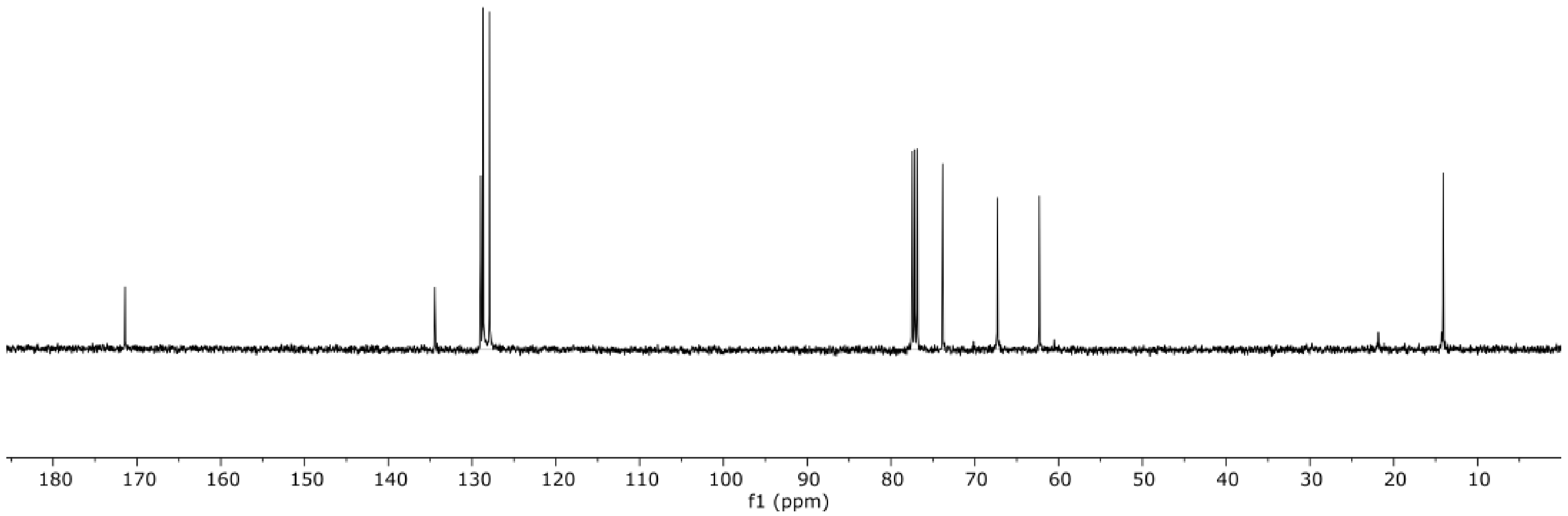
180
170
160
150
140
130
120
110
100
90
80
70
60
50
40
30
20
10
f1 (ppm)

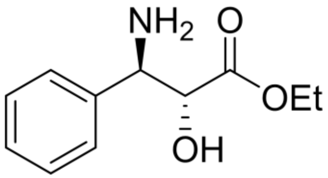
NH2
O
OEt
OH

4a

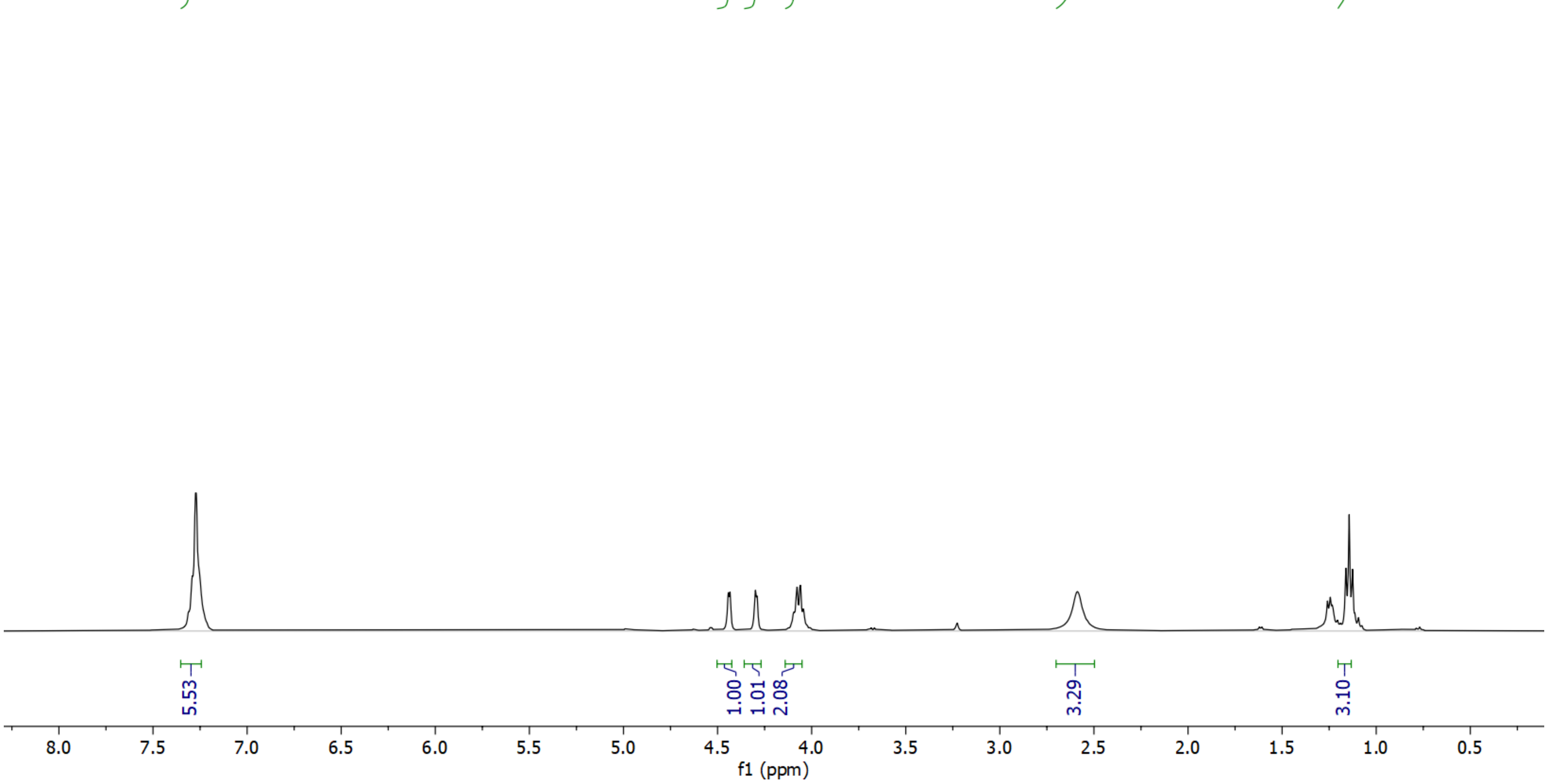
5.53
1.00
1.01
2.08
3.29
3.10
8.0
7.5
7.0
6.5
6.0
5.5
5.0
4.5
4.0
3.5
3.0
2.5
2.0
1.5
1.0
0.5
f1 (ppm)

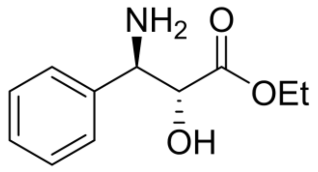

NH2
O
OEt
OH

4a

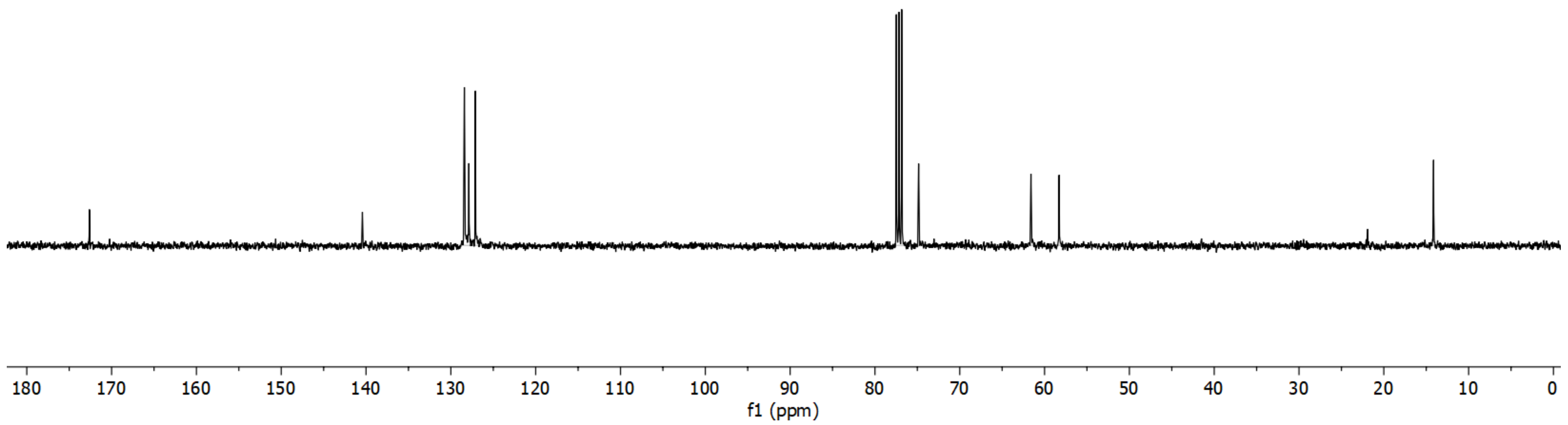

180
170
160
150
140
130
120
110
100
90
80
70
60
50
40
30
20
10
0
f1 (ppm)

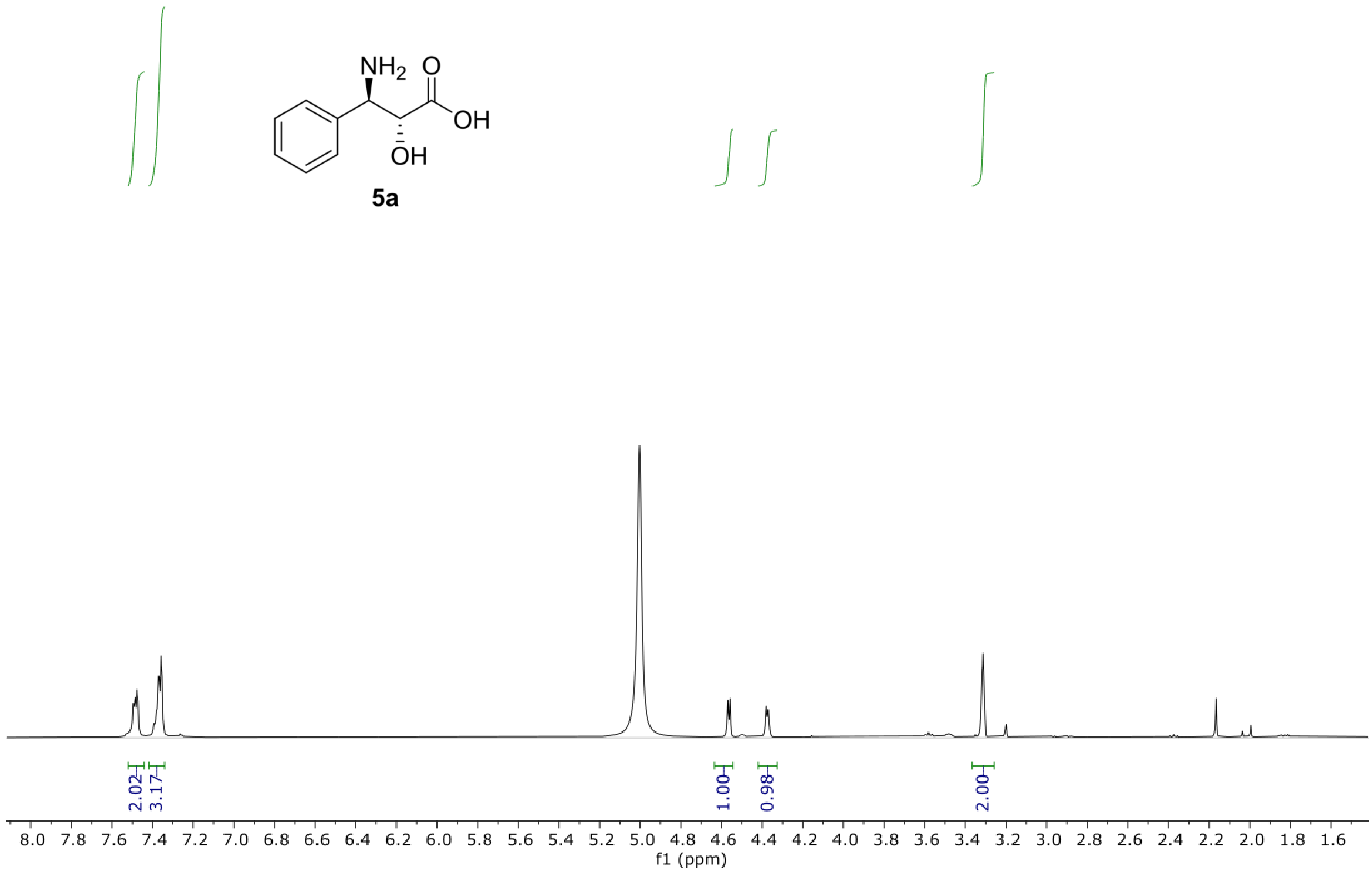

5a

2.02
3.17
1.00
0.98
2.00
8.0 7.8 7.6 7.4 7.2 7.0 6.8 6.6 6.4 6.2 6.0 5.8 5.6 5.4 5.2 5.0 4.8 4.6 4.4 4.2 4.0 3.8 3.6 3.4 3.2 3.0 2.8 2.6 2.4 2.2 2.0 1.8 1.6
f1 (ppm)

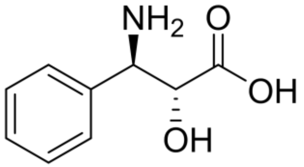
NH2
O
OH
OH

5a

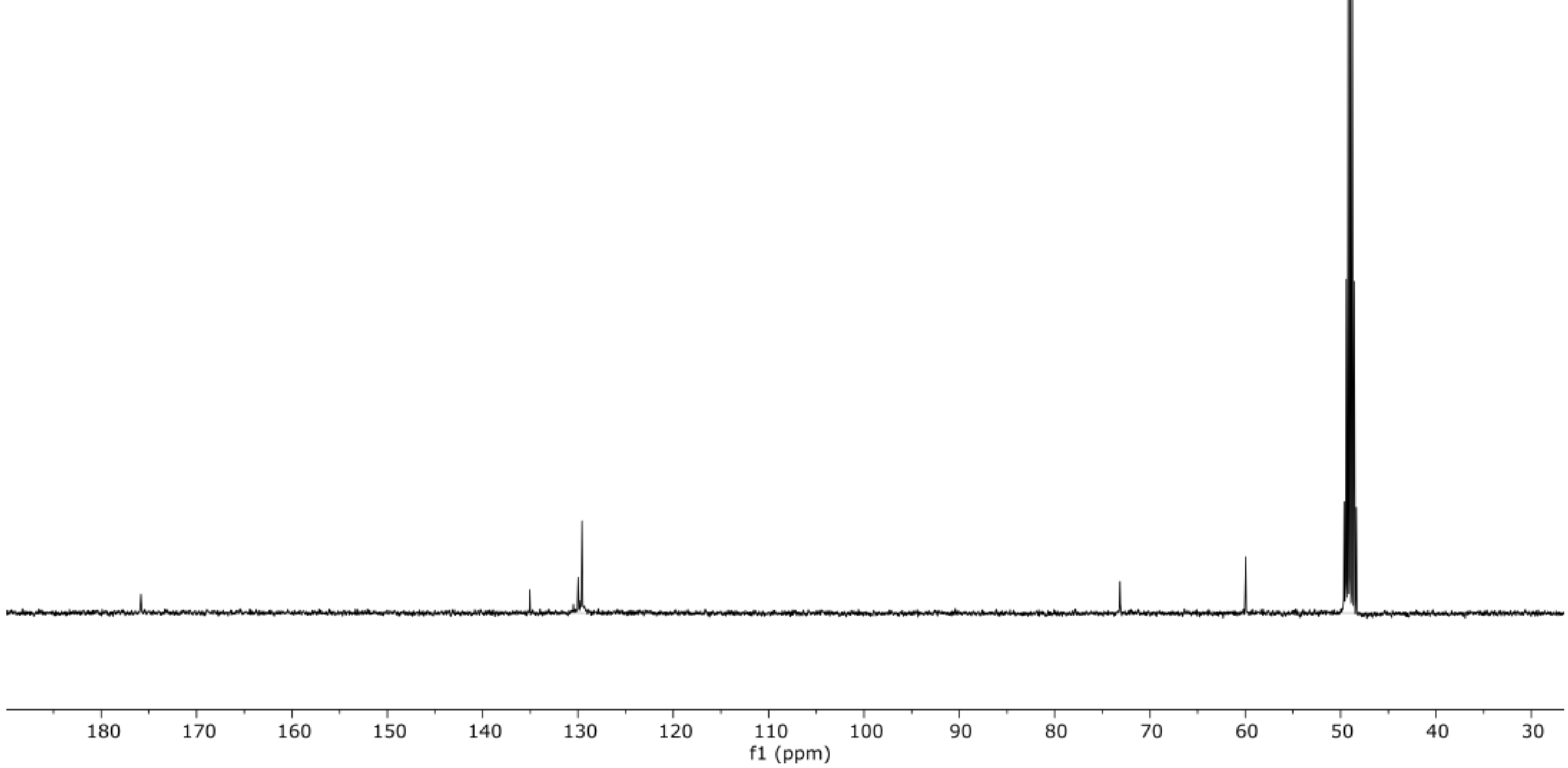
180
170
160
150
140
130
120
110
100
90
80
70
60
50
40
30
f1 (ppm)

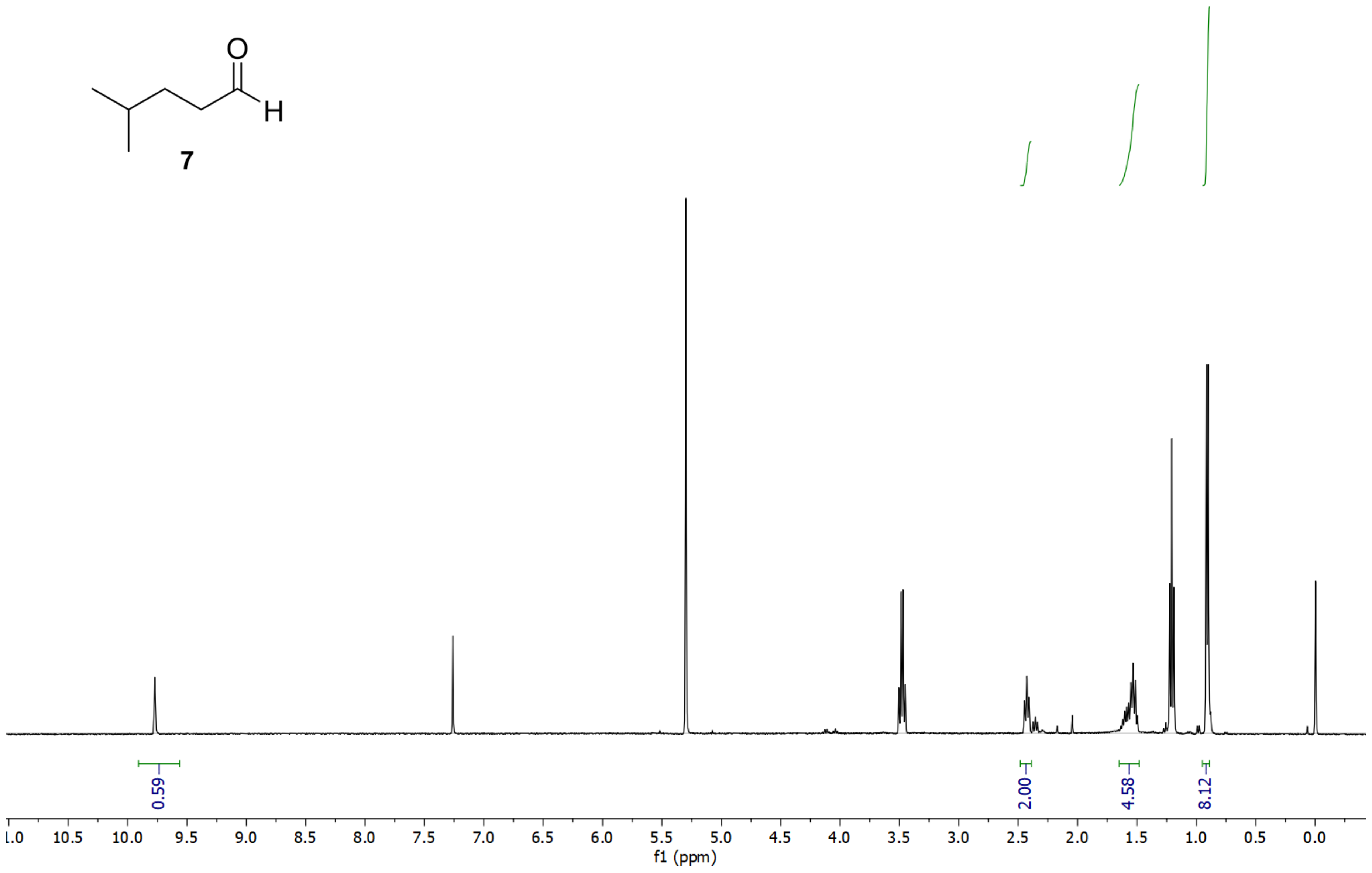
O
H
7
0.59
2.00
4.58
8.12
1.0
10.5
10.0
9.5
9.0
8.5
8.0
7.5
7.0
6.5
6.0
5.5
5.0
4.5
4.0
3.5
3.0
2.5
2.0
1.5
1.0
0.5
0.0
f1 (ppm)

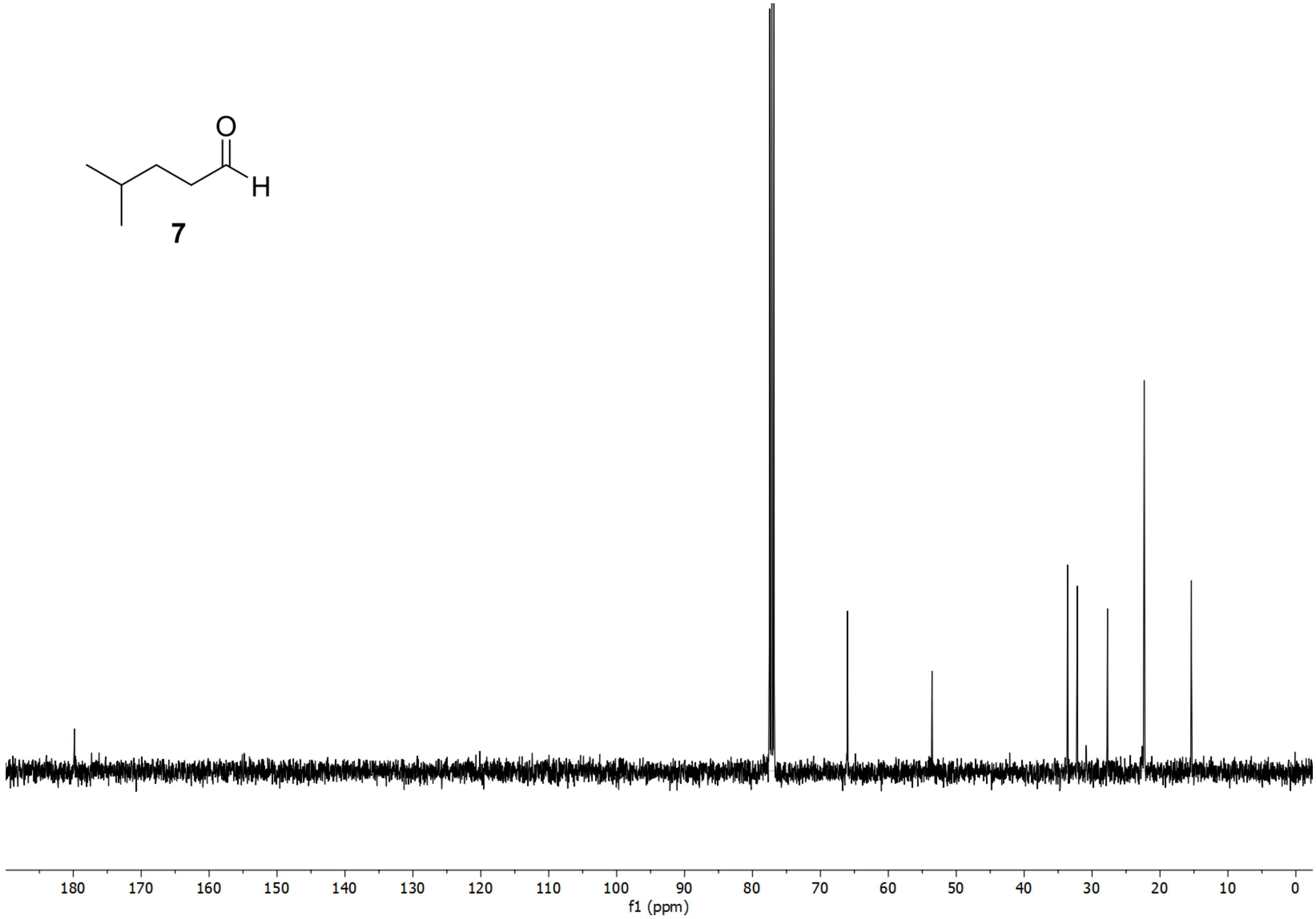

7

180
170
160
150
140
130
120
110
100
90
80
70
60
50
40
30
20
10
0
f1 (ppm)

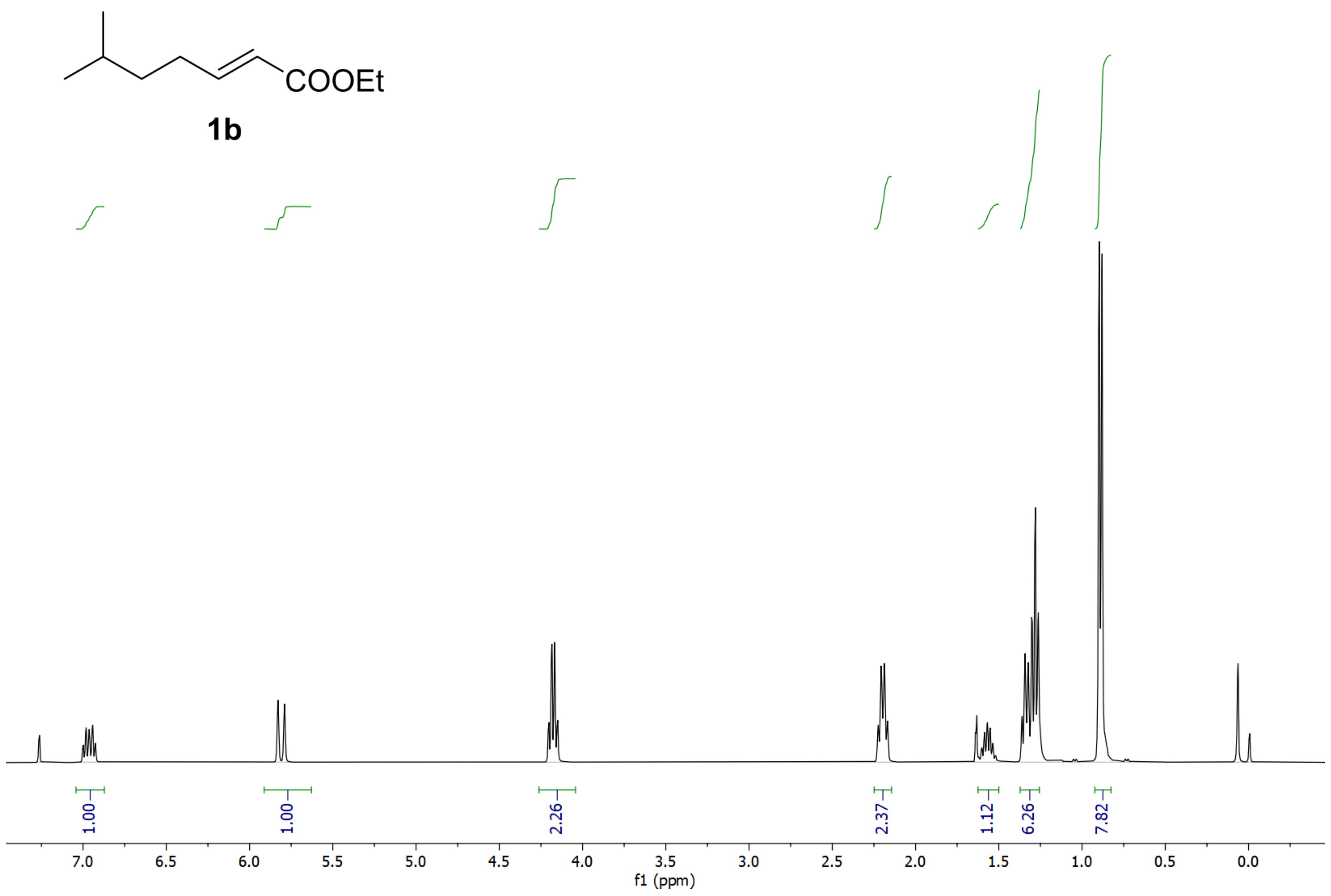

COOEt
1b
1.00
1.00
2.26
2.37
1.12
6.26
7.82
7.0
6.5
6.0
5.5
5.0
4.5
4.0
3.5
3.0
2.5
2.0
1.5
1.0
0.5
0.0
f1 (ppm)

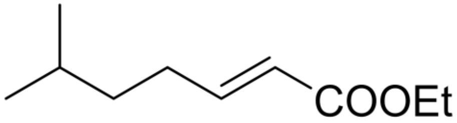
COOEt

1b

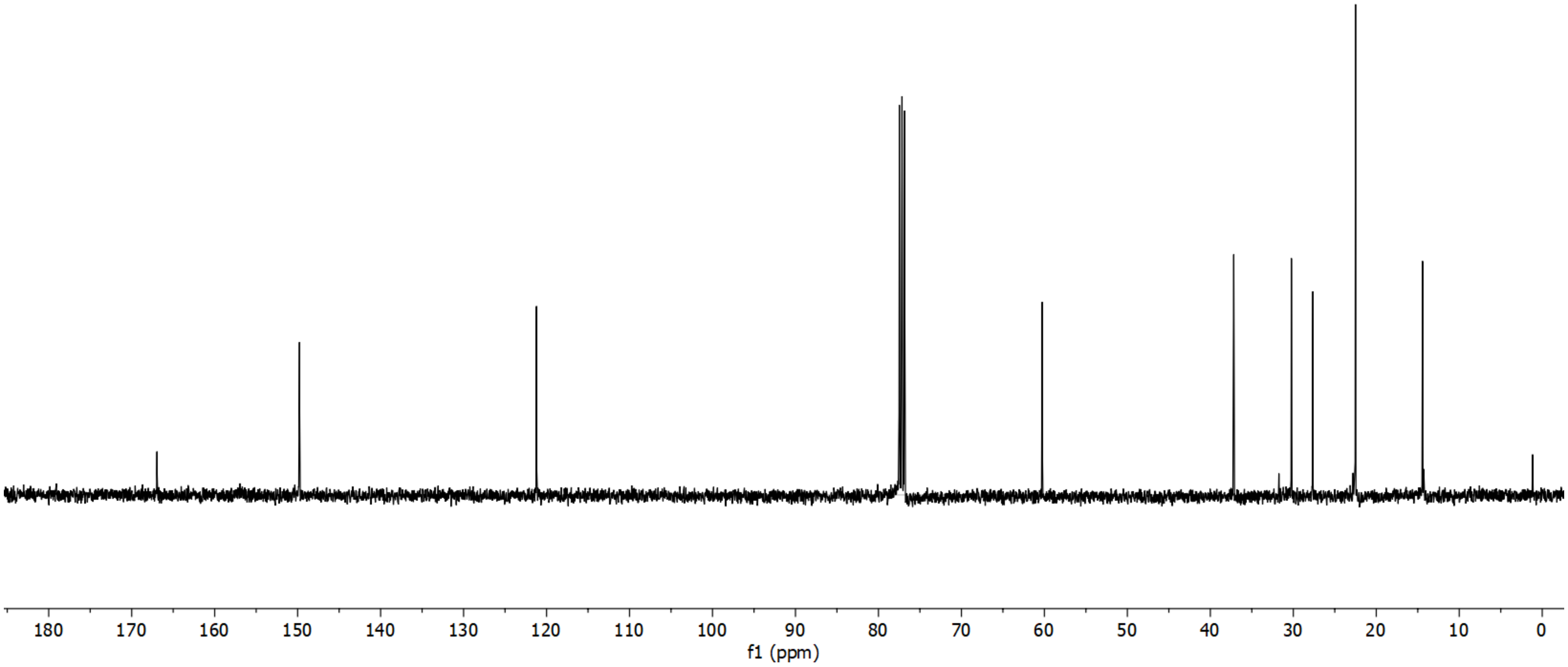
180
170
160
150
140
130
120
110
100
90
80
70
60
50
40
30
20
10
0
f1 (ppm)

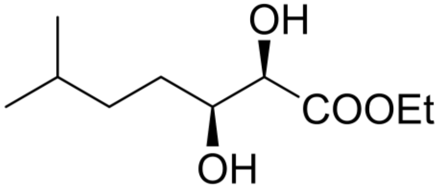
OH
COOEt
OH

2b

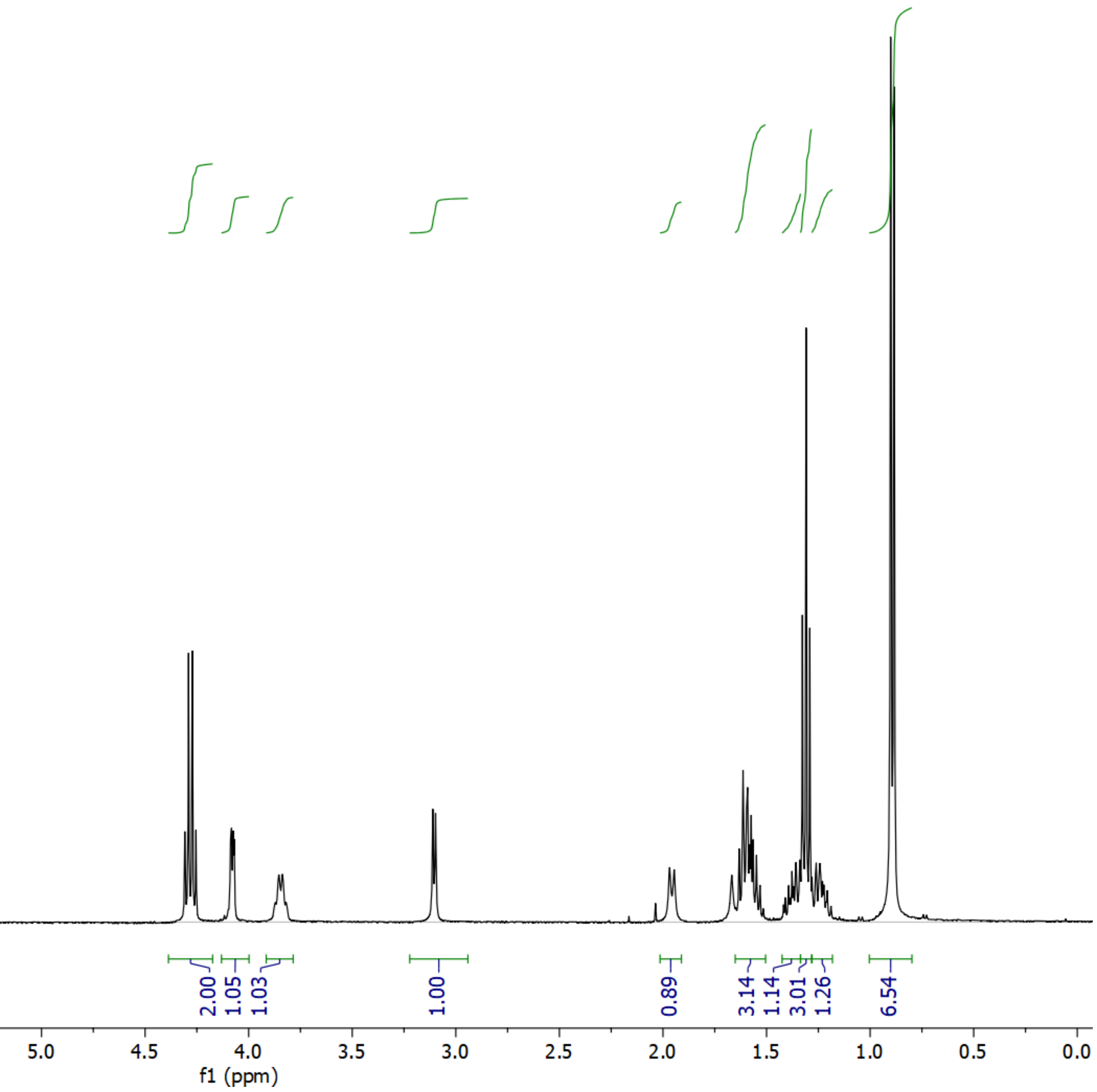
2.00
1.05
1.03
1.00
0.89
3.14
1.14
3.01
1.26
6.54
8.0
7.5
7.0
6.5
6.0
5.5
5.0
4.5
4.0
3.5
3.0
2.5
2.0
1.5
1.0
0.5
0.0
f1 (ppm)

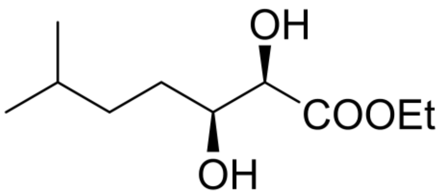
OH
COOEt
OH

2b

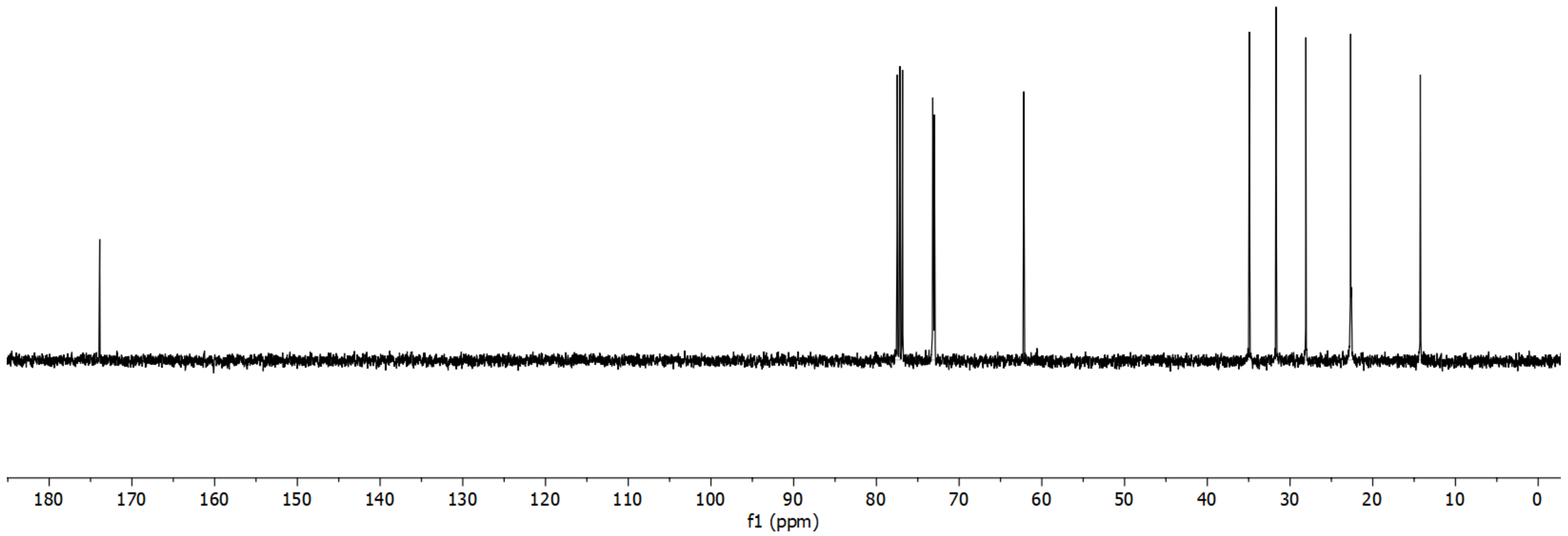
180
170
160
150
140
130
120
110
100
90
80
70
60
50
40
30
20
10
0
f1 (ppm)

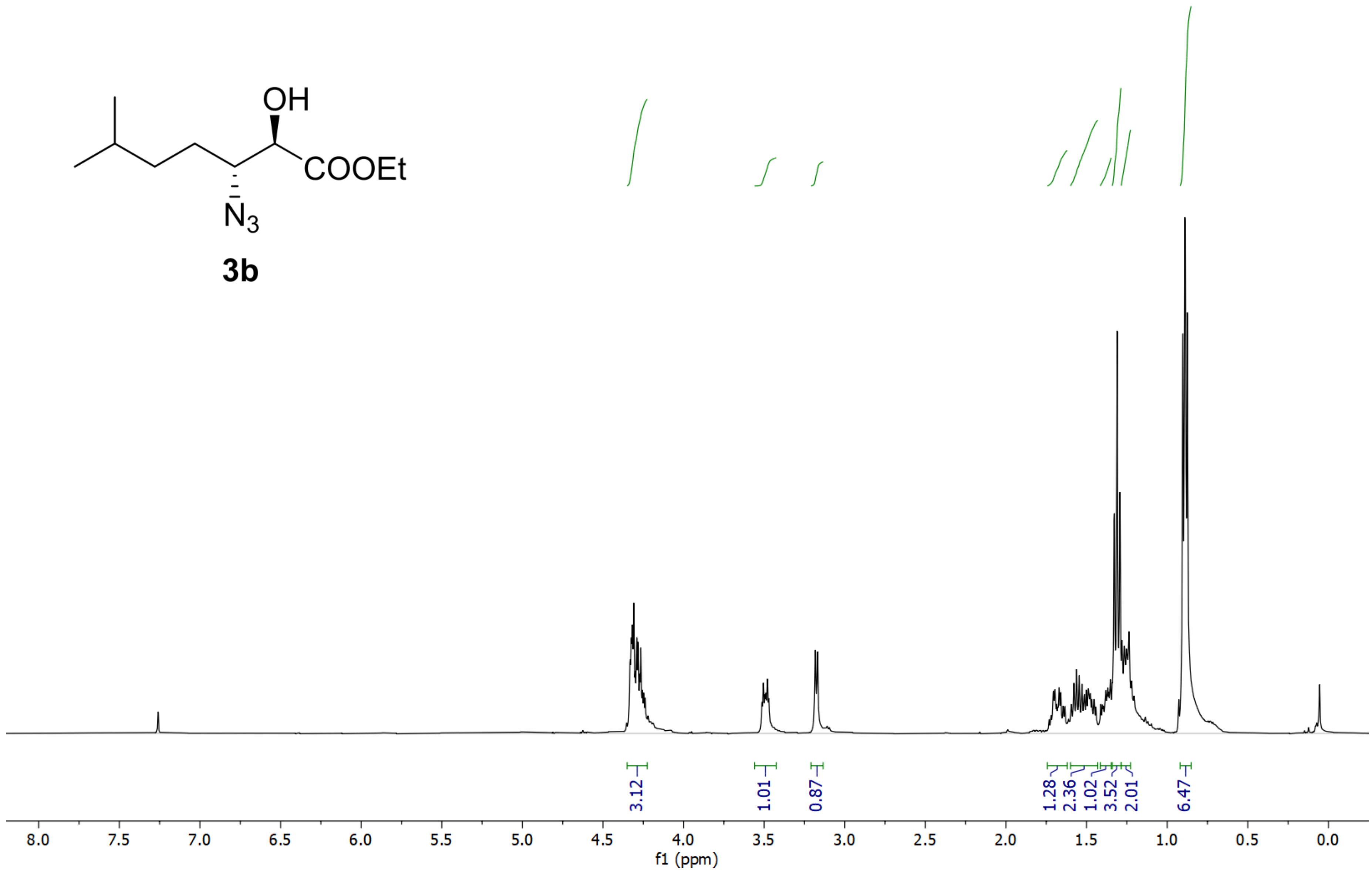
OH
COOEt
N_3
3b
3.12
1.01
0.87
1.28
2.36
1.02
3.52
2.01
6.47
8.0
7.5
7.0
6.5
6.0
5.5
5.0
4.5
4.0
3.5
3.0
2.5
2.0
1.5
1.0
0.5
0.0
f1 (ppm)

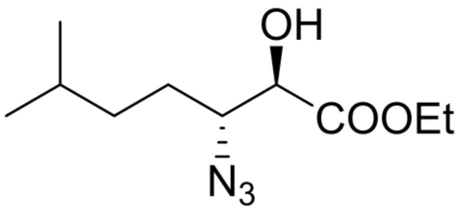
OH
COOEt
N3

3b

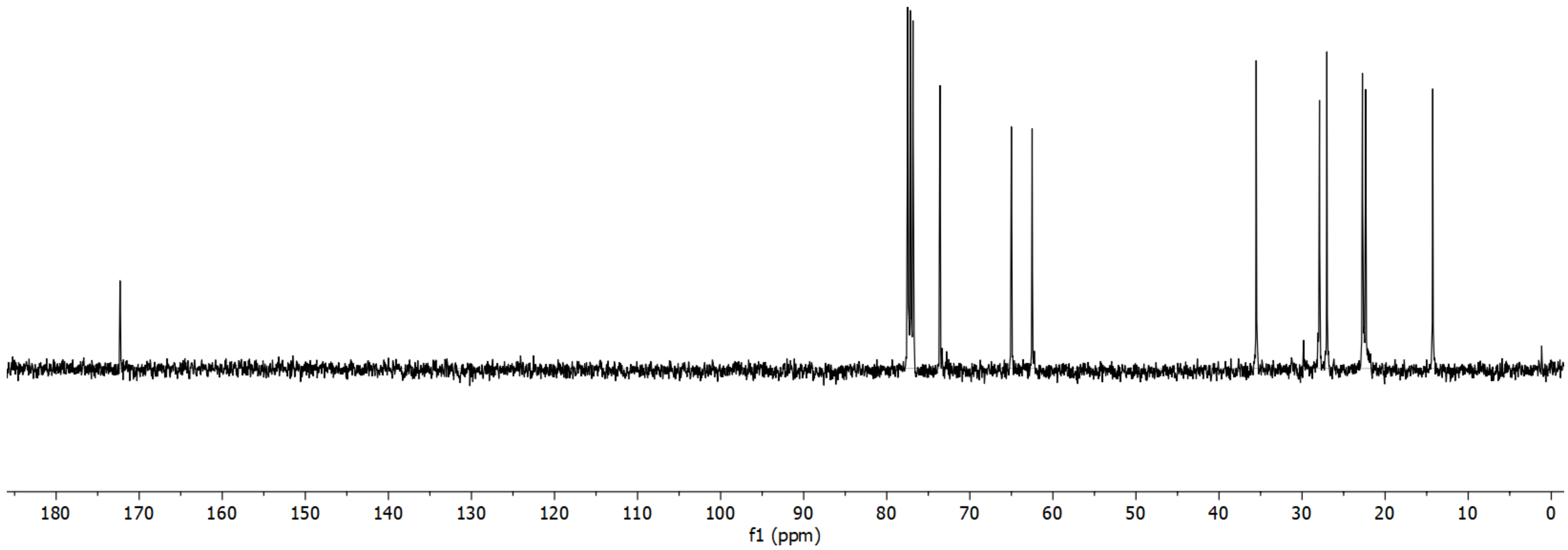
180
170
160
150
140
130
120
110
100
90
80
70
60
50
40
30
20
10
0
f1 (ppm)

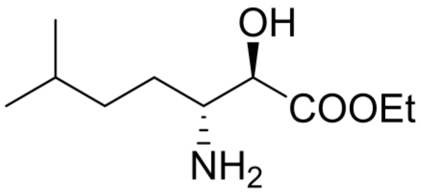
OH
COOEt
NH2

4b

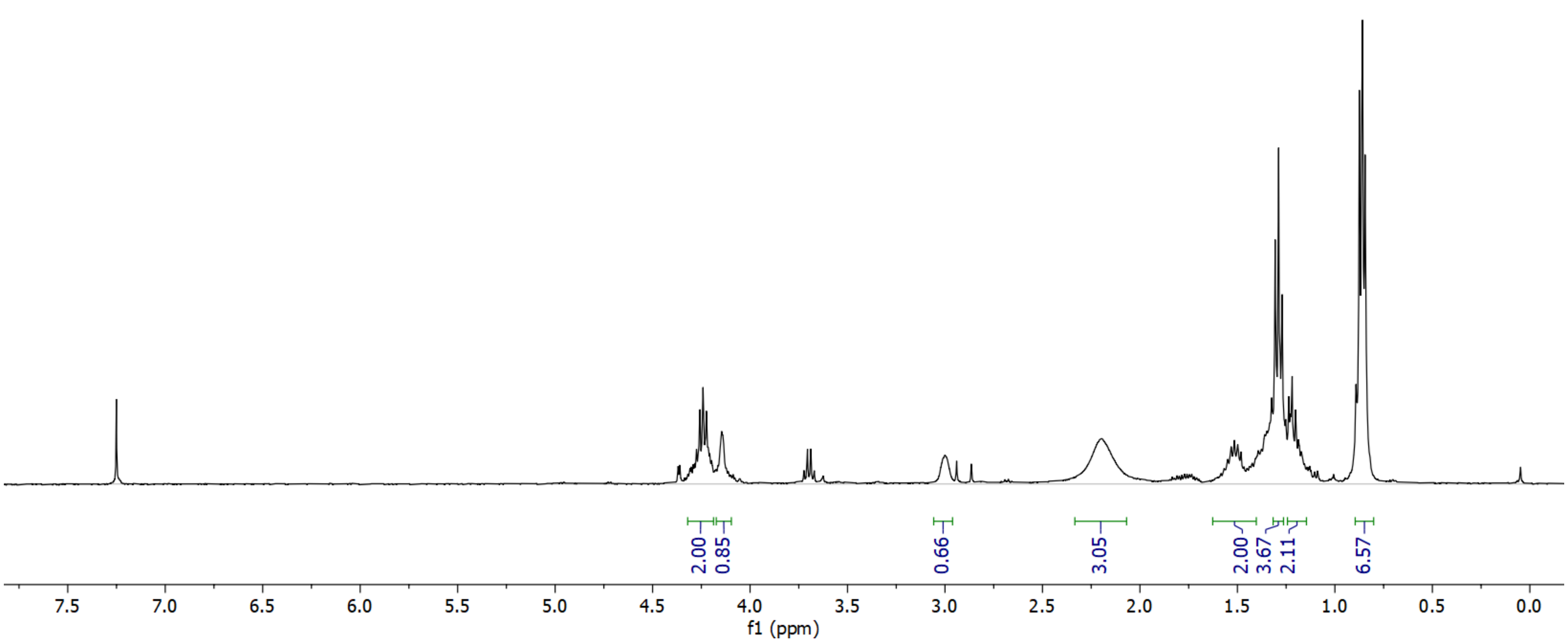
2.00
0.85
0.66
3.05
2.00
3.67
2.11
6.57
7.5
7.0
6.5
6.0
5.5
5.0
4.5
4.0
3.5
3.0
2.5
2.0
1.5
1.0
0.5
0.0
f1 (ppm)

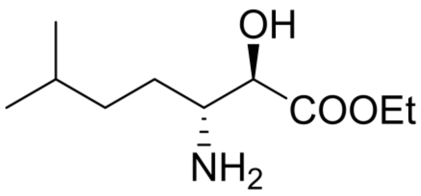
OH
COOEt
NH_2

4b

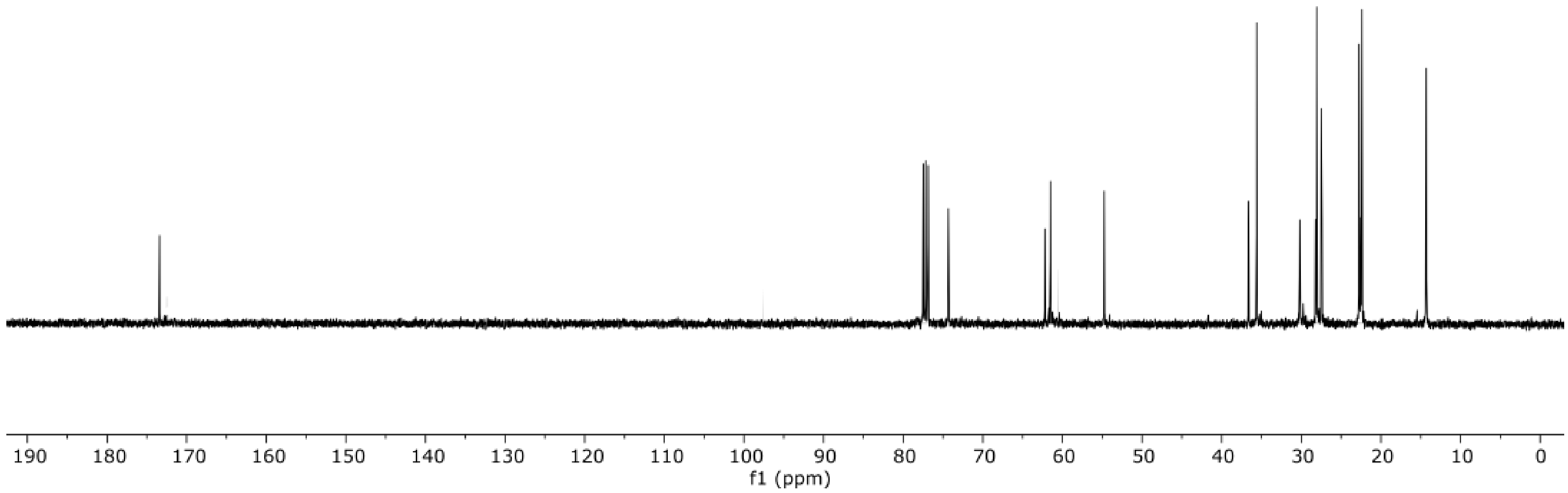
190 180 170 160 150 140 130 120 110 100 90 80 70 60 50 40 30 20 10 0
f1 (ppm)

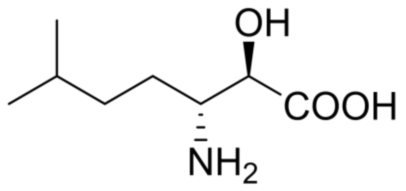
OH
COOH
NH2

AHMHA

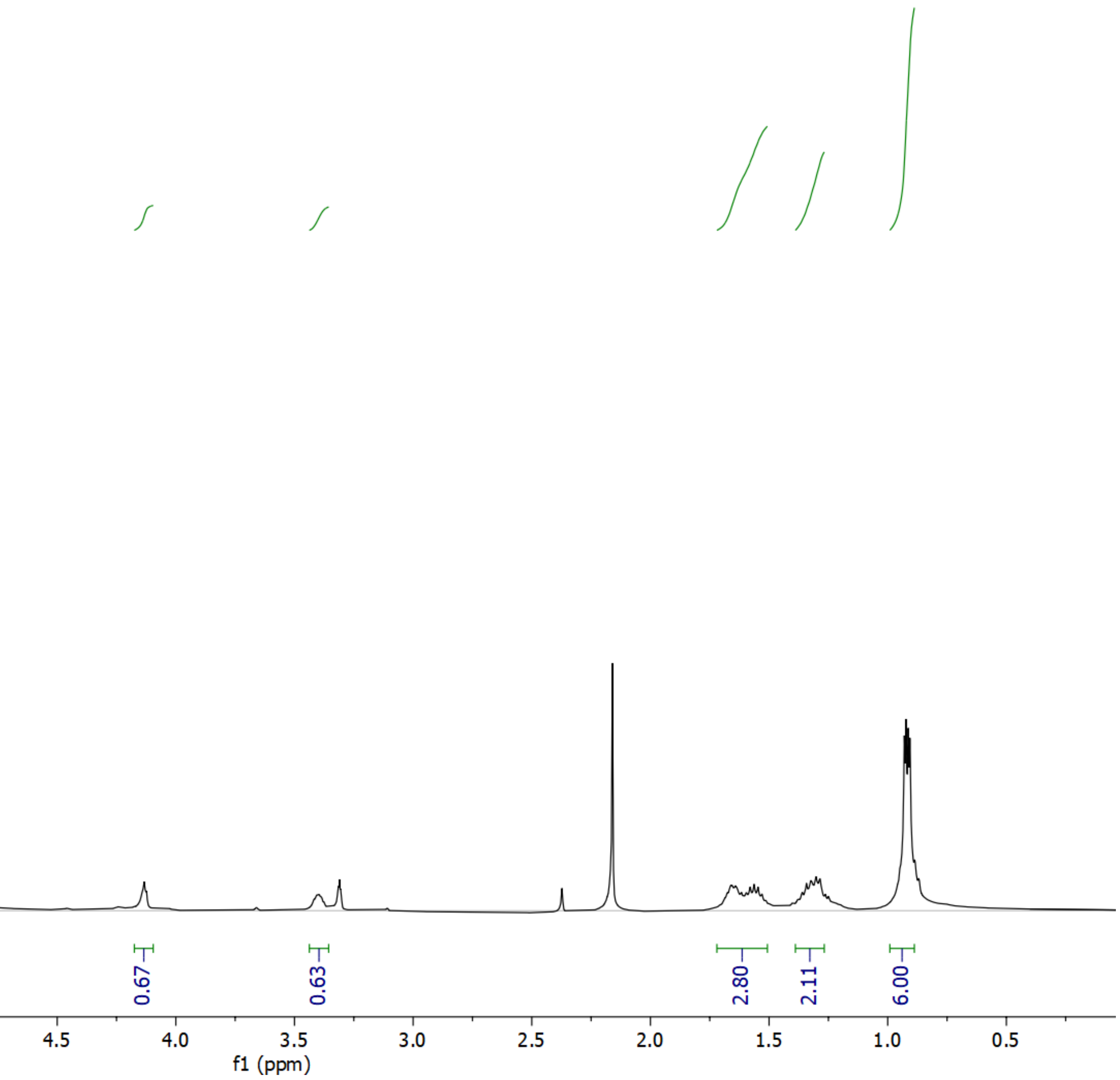
0.67
0.63
2.80
2.11
6.00
7.0
6.5
6.0
5.5
5.0
4.5
4.0
3.5
3.0
2.5
2.0
1.5
1.0
0.5
f1 (ppm)

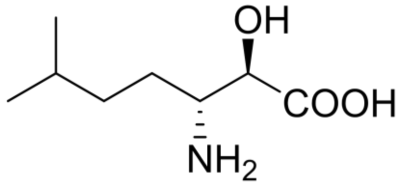
OH
COOH
NH_2

AHMHA

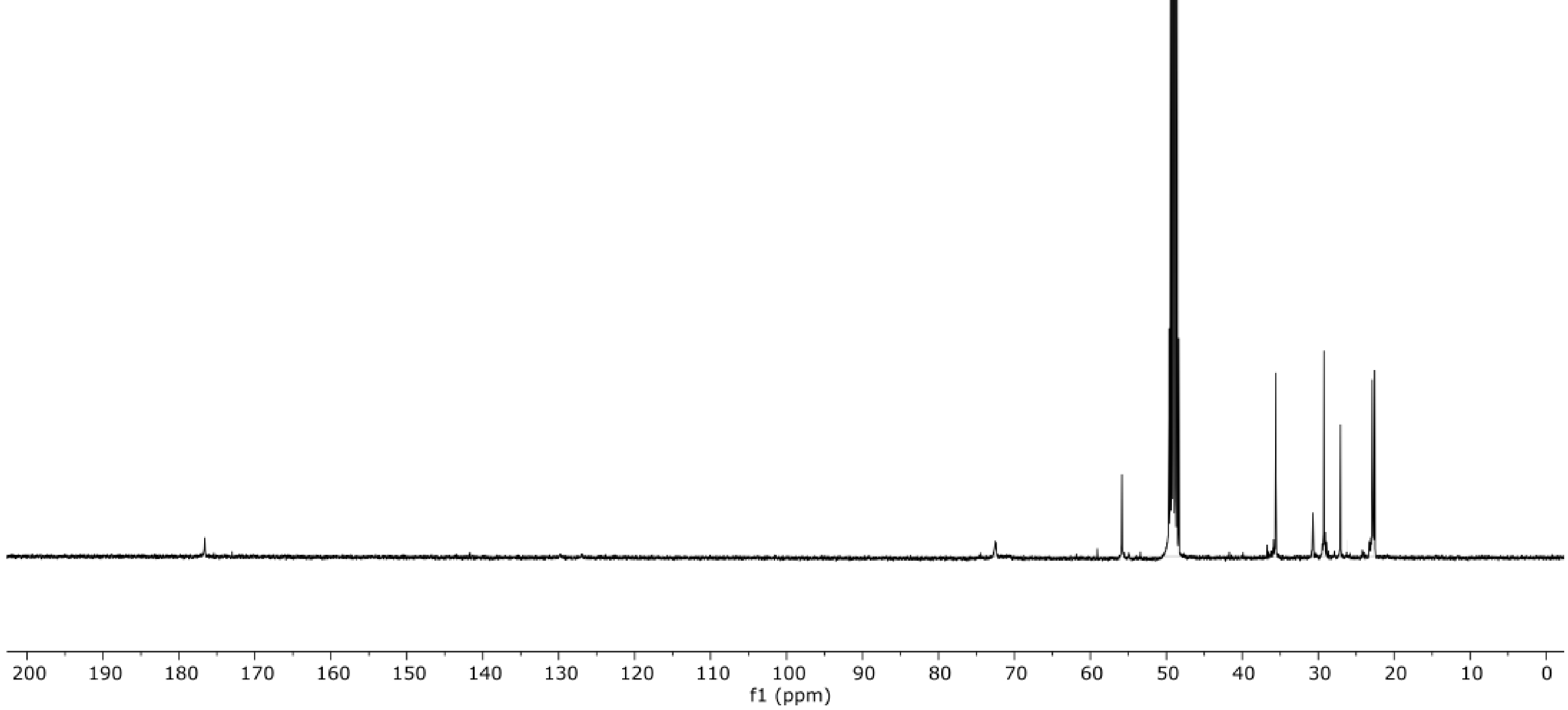
200
190
180
170
160
150
140
130
120
110
100
90
80
70
60
50
40
30
20
10
0
f1 (ppm)

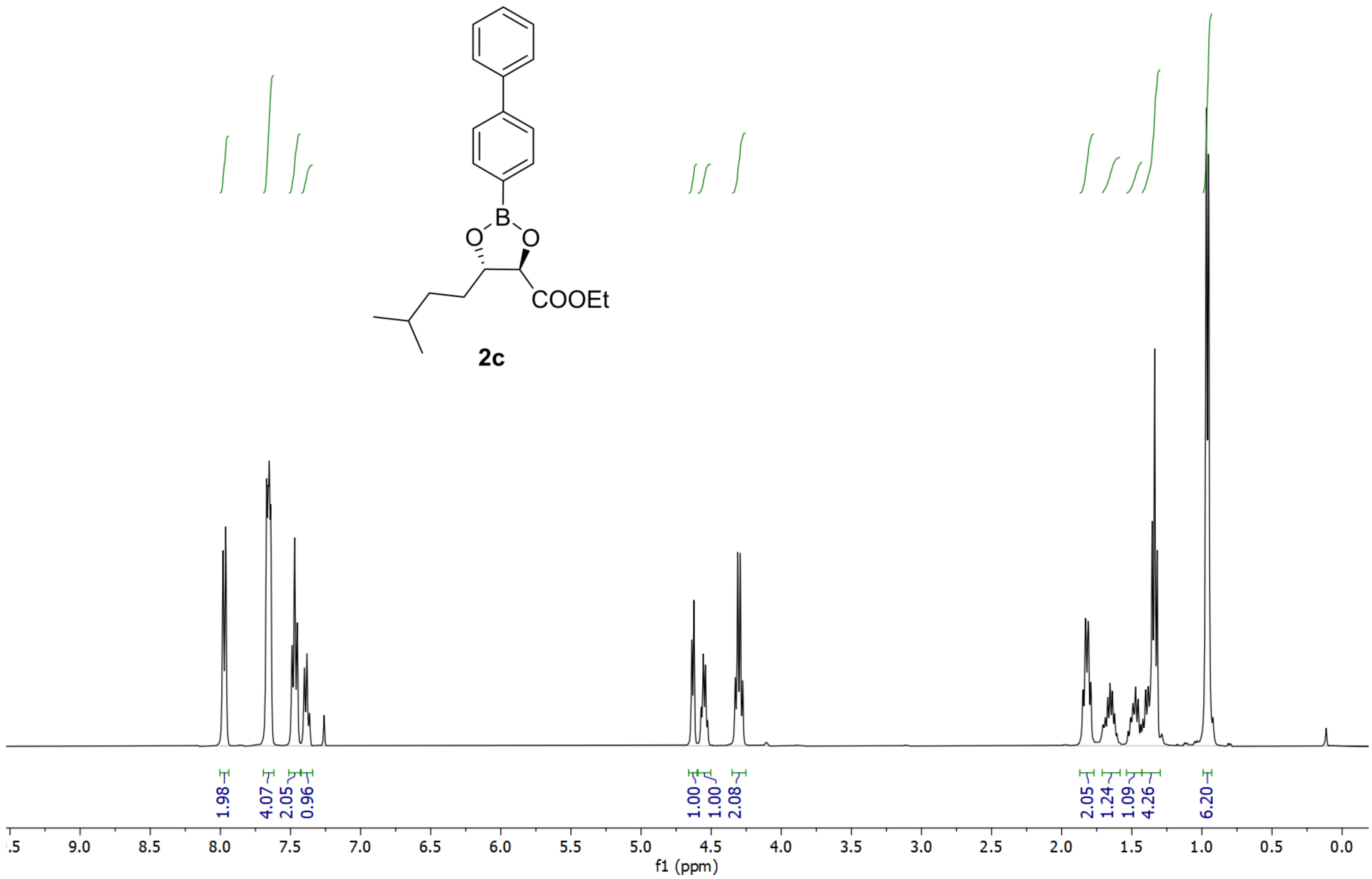
COOEt
2c
1.98
4.07
2.05
0.96
1.00
1.00
2.08
2.05
1.24
1.09
4.26
6.20
9.0 8.5 8.0 7.5 7.0 6.5 6.0 5.5 5.0 4.5 4.0 3.5 3.0 2.5 2.0 1.5 1.0 0.5 0.0
f1 (ppm)

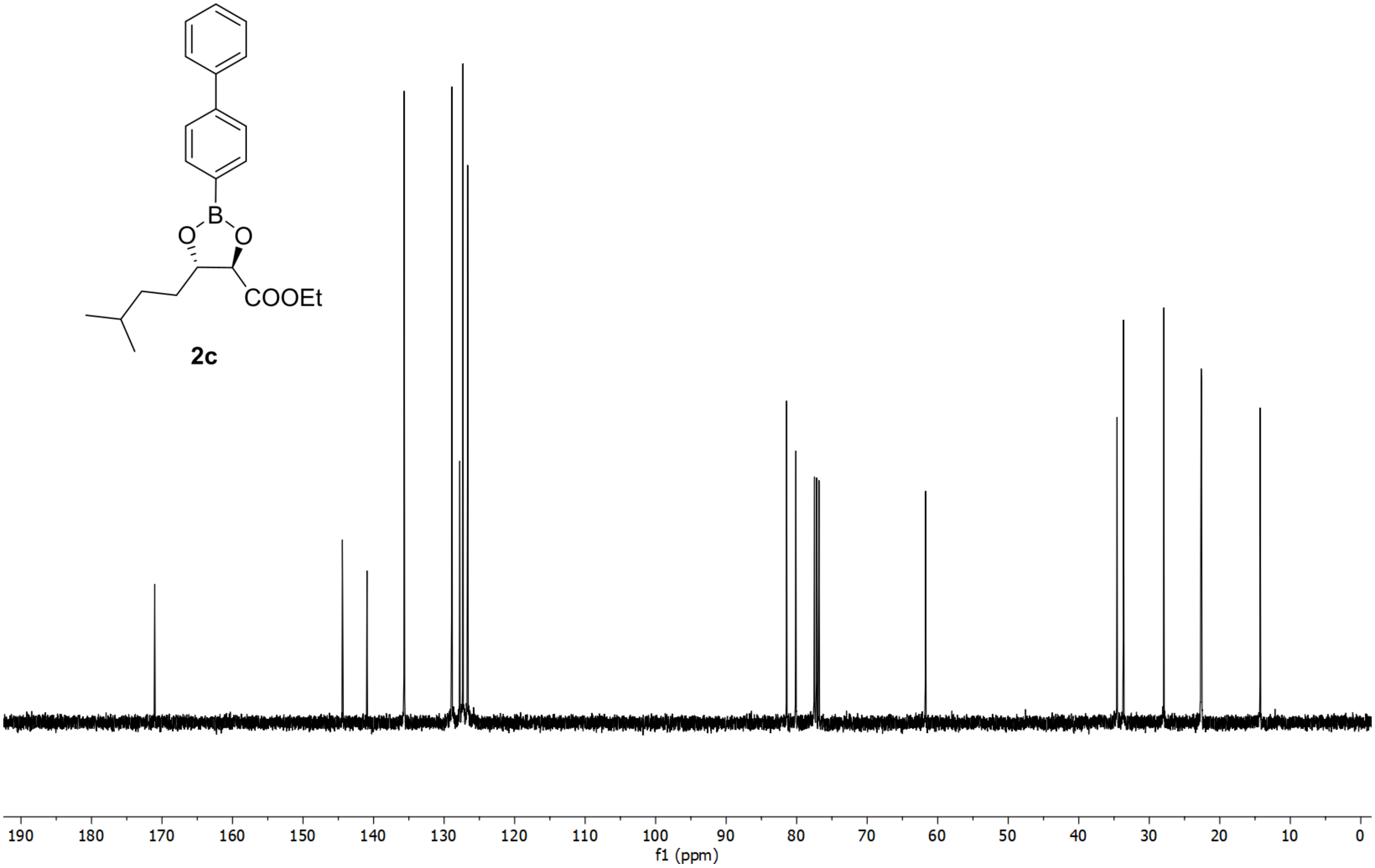
B
O
O
COOEt
190 180 170 160 150 140 130 120 110 100 90 80 70 60 50 40 30 20 10 0
f1 (ppm)

2c
